# Periodontal disease and cardiovascular disease: umbrella review

**DOI:** 10.1186/s12903-024-04907-1

**Published:** 2024-10-28

**Authors:** Heber Isac Arbildo-Vega, Fredy Hugo Cruzado-Oliva, Franz Tito Coronel-Zubiate, Joan Manuel Meza-Málaga, Sara Antonieta Luján-Valencia, Eduardo Luján-Urviola, Adriana Echevarria-Goche, Carlos Alberto Farje-Gallardo, Tania Belú Castillo-Cornock, Katherine Serquen-Olano, Tania Padilla-Cáceres, Luz Caballero-Apaza, Rubén Aguirre-Ipenza

**Affiliations:** 1 Faculty of Dentistry, Dentistry School, Universidad de San Martín de Porres, Chiclayo, 14012 Perú; 2 Faculty of Human Medicine, Human Medicine School, Universidad de San Martín de Porres, Chiclayo, 14012 Perú; 3 Faculty of Stomatology, Stomatology School, Universidad Nacional de Trujillo, Trujillo, 13001 Perú; 4 Faculty of Health Sciences, Stomatology School, Universidad Nacional Toribio Rodríguez de Mendoza de Amazonas, Chachapoyas, 01001 Perú; 5 Faculty of Medicine, Medicine School, Universidad Católica de Santa María, Arequipa, 04013 Perú; 6 Postgraduate School, Universidad Católica de Santa María, Arequipa, 04013 Perú; 7 Faculty of Dentistry, Dentistry School, Universidad Católica de Santa María, Arequipa, 04013 Perú; 8 Faculty of Dentistry, Universidad Andina Néstor Cáceres Velásquez, Juliaca, 21104 Perú; 9 Faculty of Stomatology, Universidad Peruana Cayetano Heredia, Lima, 15102 Perú; 10 Department of Dentistry, Dentistry School, Universidad Norbert Wiener, Lima, 15046 Perú; 11 Faculty of Health Sciences, Stomatology School, Universidad Señor de Sipán, Chiclayo, 14000 Perú; 12 Department of General Dentistry, Dentistry School, Universidad Nacional del Altiplano, Puno, 21001 Perú; 13 Research Institute in Environmental Sciences, Health and Biodiversity - IICASB, Universidad Nacional del Altiplano, Puno, 21001 Perú; 14 Department of Nursing, School of Nursing, Universidad Nacional del Altiplano, Puno, 21001 Perú; 15 Faculty of Health Sciences, Universidad Continental, Lima, 15046 Perú

**Keywords:** Periodontitis, Tooth loss, Inflammation, Noncommunicable diseases, Public health, Review

## Abstract

**Background:**

Periodontal disease (PD) is an infectious and inflammatory condition that affects the tissues surrounding and supporting the teeth. It has been suggested that PD may be associated with cardiovascular disease (CVD), one of the leading causes of mortality worldwide. Our study aimed to investigate the association between PD and CVD through an umbrella review.

**Methods:**

A comprehensive search was conducted until April 2024 across various electronic databases, including PubMed, Cochrane Library, Scopus, SciELO, Web of Science, Google Scholar, ProQuest Dissertations and Theses, and OpenGrey. Systematic reviews with or without meta-analysis were considered for inclusion, without any limitations on time or language, provided they examined primary studies linking PD with CVD. The AMSTAR-2 tool was employed to assess the quality and overall confidence of the included studies.

**Results:**

After the initial search, a total of 516 articles were identified. Following the application of selection criteria, 41 articles remained for further consideration. All these studies indicated an association between PD and CVD, with odds ratios and risk ratios ranging from 1.22 to 4.42 and 1.14 to 2.88, respectively.

**Conclusions:**

Systematic reviews with high overall confidence support the association between PD, tooth loss, and cardiovascular diseases. However, it is crucial to interpret these results with caution due to methodological limitations. The potential public health relevance justifies preventive and corrective oral health strategies. Additionally, the need for rigorous future research is highlighted to strengthen the evidence and guide effective public health strategies.

**Supplementary Information:**

The online version contains supplementary material available at 10.1186/s12903-024-04907-1.

## Background

Periodontal disease (PD) is a non-communicable infectious and inflammatory condition that arises due to the disruption of the balance between the commensal oral microbiome and the host immune response, leading to tissue deterioration and hampering the effective elimination of bacteria [1, 2]. PD encompasses a range of conditions that affect the supporting structures of the teeth, including gingivitis and periodontitis. Gingivitis is the mildest and reversible form, characterized by gum inflammation without destruction of the alveolar bone [3]. In contrast, periodontitis is a more advanced form that can lead to the destruction of the periodontal ligament, cementum, and alveolar bone, resulting in tooth loss if not adequately treated [4].

The anatomical structures involved in periodontitis include the gums, periodontal ligament, cementum, and alveolar bone [4]. The gums act as a protective barrier surrounding the teeth and help prevent bacterial invasion. The periodontal ligament, composed of collagen fibers, connects the tooth to the alveolar bone, providing support and cushioning during mastication. The cementum covers the tooth roots and serves as an anchoring site for the periodontal ligament fibers, while the alveolar bone offers the structural foundation for the teeth [5]. Inflammation in these structures can lead to progressive tissue destruction [6].

Although periodontitis is mostly an infection caused by gram-negative bacteria, evidence suggests that degradation of periodontal tissue is influenced by the host immune response [7]. The etiology of periodontitis is multifactorial, involving both microbial and non-microbial factors. Pathogens such as *Porphyromonas gingivalis* and *Tannerella forsythia* play a crucial role by evading the host immune response and promoting chronic inflammation [8]. The release of pro-inflammatory cytokines such as IL-1β and TNF-α by the host exacerbates periodontal tissue destruction [9]. Additionally, genetic predispositions, systemic conditions such as diabetes mellitus, and lifestyle factors such as smoking are key determinants in the development and progression of periodontitis [10].

While PD can manifest in various clinical forms, its diagnosis is based on the assessment of the severity and rate of disease progression [11]. Treatment modalities for periodontitis are divided into non-surgical and surgical approaches. Non-surgical periodontal treatment, such as scaling and root planing, focus on the mechanical removal of dental plaque and calculus from the tooth surfaces and subgingival areas, thereby reducing bacterial load and inflammation. When these treatments are insufficient, surgical interventions such as guided tissue regeneration (GTR) and bone grafting are employed to restore damaged periodontal structures [12, 13]. The choice of treatment depends on the severity of the disease and the patient's response to initial therapy [14].

Determining the global prevalence of PD is complicated due to case definitions and the heterogeneity of the studies carried out [15]. Ikeda et al. [16] revealed that both healthy individuals and patients with periodontitis presented differences not only in microbial composition, but also in genetic expression and metabolic pathways. The bidirectional nature of the relationship between bacteria and the host is well-known bacteria can induce inflammation, while inflammation can alter the environment, leading to changes in the microbiome composition [1].

In recent research, it has been confirmed that the oral cavity houses the second most extensive microbiota in the human body, with approximately 500 different bacterial species identified so far. These bacteria play an essential role in the regulation of human health and the appearance of various pathologies [17].

Oral dysbiosis is not only associated with periodontal disorders, but also with metabolic disorders, such as cardiovascular diseases (CVDs) [18]. It is important to highlight that currently, the mortality and morbidity associated with CVDs represent a major challenge for society [19]. Ischemic heart disease, stroke, and hypertension (which can lead to heart failure) are the leading causes of CVD-related death [20].

Therefore, both PD and CVD are among the most common conditions worldwide that generate chronic inflammation with degenerative characteristics [21]. This chronic inflammation is manifested by an increase in the levels of inflammatory cytokines, which leads to a weakening of the function of the immune system, thus increasing the risk of atherosclerosis and insulin resistance, which are primary factors in the development of CVD [22].

Currently, systematic review(s) (SR) on the association of PD with CVD have been published in the scientific literature. However, a current general synthesis and evaluation, covering all SR on this possible association, would be of great value to better understand this relationship and its impact on public health. Such a synthesis would allow for the identification of consistent patterns, areas of uncertainty, and potential gaps in research. In addition, it would help establish evidence-based recommendations for the prevention and management of both conditions in a comprehensive manner.

Hence, the objective of this umbrella review was to consolidate the existing evidence and address the following precise inquiry: "What is the current understanding regarding the relationship between PD and CVD?" Additionally, how overall confidence are SR in evaluating this topic?

## Methods

### Protocol and registration

A protocol was developed in accordance with the Preferred Reporting Items for Systematic Reviews and Meta-Analysis Protocols (PRISMA-P) guidelines [23] and registered in the Prospective Registry of Systematic Reviews (PROSPERO) [24] under the registration number CRD42024521095. The study adheres to the reporting standards outlined in the Preferred Reporting Items for Overview of Systematic Reviews Checklist (PRIO-harms) [25]. Ethical approval was deemed unnecessary for this umbrella review.

The research question was formulated using the PECO framework (population, exposure, comparison, and outcomes) as follows:

- Population: individuals of all ages.
- Exposure: individuals with CVD.
- Comparison: individuals without CVD.
- Outcomes: association with PD.

### Eligibility criteria and results of interest

The eligible studies comprised SR with or without meta-analysis, without limitations on publication date or language, that investigated primary studies exploring the association between PD and CVD. Excluded were literature or narrative reviews, rapid reviews, intervention studies, observational studies, preclinical and basic research, abstracts, commentaries, case reports, protocols, personal opinions, letters, and posters.

### Sources of information, search strategy, and additional search for primary studies

On April 12th, 2024, an electronic search was conducted across five databases, including PubMed, Cochrane database, SciELO, Web of Science, and Scopus. Grey literature was explored through Google Scholar, Proquest Dissertations and Theses, and OpenGrey. Furthermore, reference lists of the included studies were screened. Retrieved articles were managed using reference management software (Zotero® 6.0, Center for History and New Media, Fairfax, Virginia, USA), and duplicate entries were eliminated. The search strategies implemented for each database are detailed in Table 1.

**Table 1:** Search strategy for each search engine

Database	Strategy		Number of Studies
PubMed	#1	(("Periodontal Disease") OR ("furcation defect") OR ("gingival disease") OR ("periodontitis") OR ("tooth migration") OR ("tooth mobility") OR ("tooth loss")) AND (("Cardiovascular Disease") OR ("coronary heart disease") OR ("coronary artery disease") OR ("myocardial infarction") OR ("coronary arteriosclerosis") OR ("heart attack") OR ("heart failure") OR ("heart decompensation") OR ("atrial fibrillation") OR ("sudden cardiac death") OR ("arrhythmia") OR ("cardiomyopathy") OR ("hypertrophic cardiomyopathy") OR ("dilated cardiomyopathy"))	67
Cochrane	#1	MeSH descriptor: [Periodontal Diseases] explode all trees	35
	#2	MeSH descriptor: [Furcation Defects] explode all trees	
	#3	MeSH descriptor: [Gingival Diseases] explode all trees	
	#4	MeSH descriptor: [Periodontitis] in all MeSH products	
	#5	MeSH descriptor: [Tooth Migration] explode all trees	
	#6	MeSH descriptor: [Tooth Mobility] explode all trees	
	#7	MeSH descriptor: [Tooth Loss] explode all trees	
	#8	("Periodontal Disease") OR (“furcation defect”) OR (“gingival disease”) OR ("periodontitis") OR ("tooth migration") OR ("tooth mobility") OR ("tooth loss") (Word variations have been searched)	
	#9	#1 OR #2 OR #3 OR #4 OR #5 OR #6 OR #7 OR #8	
	#10	MeSH descriptor: [Cardiovascular Diseases] explode all trees	
	#11	MeSH descriptor: [Coronary Disease] explode all trees	
	#12	MeSH descriptor: [Coronary Artery Disease] explode all trees	
	#13	MeSH descriptor: [Myocardial Infarction] explode all trees	
	#14	MeSH descriptor: [Heart Failure] explode all trees	
	#15	MeSH descriptor: [Atrial Fibrillation] explode all trees	
	#16	MeSH descriptor: [Death, Sudden, Cardiac] explode all trees	
	#17	MeSH descriptor: [Arrhythmias, Cardiac] explode all trees	
	#18	MeSH descriptor: [Cardiomyopathies] explode all trees	
	#19	MeSH descriptor: [Cardiomyopathy, Hypertrophic] explode all trees	
	#20	MeSH descriptor: [Cardiomyopathy, Dilated] explode all trees	
	#21	("Cardiovascular Disease") OR ("coronary heart disease") OR (“coronary artery disease”) OR (“myocardial infarction”) OR (“coronary arteriosclerosis”) OR (“heart attack”) OR (“heart failure”) OR (“heart decompensation”) OR (“atrial fibrillation”) OR (“sudden cardiac death”) OR ("arrhythmia") OR ("cardiomyopathy") OR (“hypertrophic cardiomyopathy”) OR (“dilated cardiomyopathy”)	
	#22	#10 OR #11 OR #12 OR #13 OR #14 OR #15 OR #16 OR #17 OR #18 OR #19 OR #20 OR #21	
	#23	MeSH descriptor: [Systematic Reviews as Topic] explode all trees	
	#24	MeSH descriptor: [Meta-Analysis as Topic] explode all trees	
	#25	("systematic review") OR ("meta-analysis") (Word variations have been searched)	
	#26	#23 OR #24 OR #25	
	#27	#9 AND #22 AND #26	
Scopus	#1	(TITLE-ABS-KEY ((("Periodontal Disease") OR ("furcation defect") OR ("gingival disease") OR ("periodontitis") OR ("tooth migration") OR ("tooth mobility") OR ("tooth loss"))) AND TITLE-ABS-KEY ((("Cardiovascular Disease") OR ("coronary heart disease") OR ("coronary artery disease") OR ("myocardial infarction") OR ("coronary arteriosclerosis") OR ("heart attack") OR ("heart failure") OR ("heart decompensation") OR ("atrial fibrillation") OR ("sudden cardiac death") OR ("arrhythmia") OR ("cardiomyopathy") OR ("hypertrophic cardiomyopathy") OR ("dilated cardiomyopathy"))) AND TITLE-ABS-KEY ((("systematic review") OR (meta-analysis)))) AND (LIMIT-TO (DOCTYPE, "re")) AND (LIMIT-TO (PUBSTAGE, "final")) AND (LIMIT-TO (SRCTYPE, "j"))	195
Web of Science	#1	(TS=("periodontitis") OR TS=("periodontal disease") OR TS=("furcation defect") OR TS=("gingival disease") OR TS=("tooth loss") OR TS=("tooth migration") OR TS=("tooth mobility")) AND (TS=("Cardiovascular Disease") OR TS=("coronary heart disease") OR TS=("coronary artery disease") OR TS=("myocardial infarction") OR TS=("coronary arteriosclerosis") OR TS=("heart attack") OR TS=("heart failure") OR TS=("heart decompensation") OR TS=("atrial fibrillation") OR TS=("sudden cardiac death") OR TS=("arrhythmia") OR TS=("cardiomyopathy") OR TS=("hypertrophic cardiomyopathy") OR TS=("dilated cardiomyopathy")) AND (TS=("systematic review") OR TS=("meta-analysis"))	166
Scielo	#1	((("Periodontal Disease") OR (“furcation defect”) OR (“gingival disease”) OR (“periodontitis”) OR (“tooth migration”) OR (“tooth mobility”) OR (“tooth loss”))) AND ((("Cardiovascular Disease") OR (“coronary heart disease”) OR (“coronary artery disease”) OR (“myocardial infarction”) OR (“coronary arteriosclerosis”) OR (“heart attack”) OR (“heart failure”) OR (“heart decompensation”) OR (“atrial fibrillation”) OR (“sudden cardiac death”) OR (“arrhythmia”) OR (“cardiomyopathy”) OR (“hypertrophic cardiomyopathy”) OR (“dilated cardiomyopathy”)))	26
Google Scholar	#1	allintitle: (("periodontal disease") OR ("periodontitis")) + ("cardiovascular disease") + (("systematic review") OR ("meta-analysis"))	12
Proquest Dissertations and Theses	#1	("Periodontal Disease" OR “gingival disease” OR “periodontitis”) AND ("Cardiovascular Disease") AND (“systematic review” OR "meta-analysis") NOT ("obesity" OR "animal" OR "in vitro" OR "diabetes" OR "caries" OR "vitamin" OR "cancer" OR "protein" OR "photodynamic" OR "implant" OR "knowledge")	15
OpenGrey	#1	(("Periodontal Disease") OR ("furcation defect") OR ("gingival disease") OR ("periodontitis") OR ("tooth migration") OR ("tooth mobility") OR ("tooth loss")) AND (("Cardiovascular Disease") OR ("coronary heart disease") OR ("coronary artery disease") OR ("myocardial infarction") OR ("coronary arteriosclerosis") OR ("heart attack") OR ("heart failure") OR ("heart decompensation") OR ("atrial fibrillation") OR ("sudden cardiac death") OR ("arrhythmia") OR ("cardiomyopathy") OR ("hypertrophic cardiomyopathy") OR ("dilated cardiomyopathy"))	0

### Data management and selection process

The articles identified were inputted into the Rayyan® Online Software, managed by the Qatar Research Institute of Computing in Doha, Qatar. The study selection process occurred in two phases: initially, two reviewers (F.C.O. and F.C.Z.) independently assessed titles and abstracts. Subsequently, phase 2 involved the independent review of full text articles by the same two reviewers. In case of any discrepancies, a third reviewer (H.A.) was consulted for resolution.

### Data collection process

Information from the studies was collected independently and in duplicate using a table previously prepared by two reviewers (F.C.O. and R.A.). The data were then cross verified, and any discrepancies were resolved by consulting the third author (H.A.). The extracted information from the selected articles included details such as authors, publication year, study design, primary study design, number of studies included in qualitative and quantitative analyses, results, main conclusions, and any mention of frameworks or methodologies used, such as PRISMA, PROSPERO, Grading of Recommendations Assessment, Development and Assessment (GRADE), and meta-analysis.

### Assessment of methodological quality, quality of evidence, and *meta*-*bias*

Two reviewers (J.M. and S.L.) independently conducted a duplicate evaluation of the methodological quality of the included SR, with a calibration of Kappa 0.85, using the AMSTAR-2 checklist (A Measurement Tool to Assess Systemic Reviews) [26]. AMSTAR-2 assesses the methodological quality of SR through 16 questions, each with three possible responses: "yes," "no," or "partially yes." The overall confidence rating of the studies, categorized as high, moderate, low, or critically low, was determined following the guidelines proposed by Shea et al. [26].

### Summary of measures

For SR without meta-analysis, we considered the summarized results from the primary studies included. However, if the SR included a meta-analysis, we focused on the results presented with odds ratio (OR), hazard ratio (HR), risk/rate ratio (RR), or mean difference (MD) to assess the association between PD and CVD.

### Summary of results

The primary outcomes of the included SR were summarized, organizing their findings into various categories related to cardiovascular health. These categories encompassed CVD, cerebrovascular disease, atherosclerotic cardiovascular disease, acute coronary syndrome, atrial fibrillation/atrial flutter, arterial stiffness, cardiac arrhythmias, carotid atherosclerosis, carotid artery calcification, coronary artery disease, carotid artery disease, cardiac death, coronary heart disease, carotid intima-media thickness/flow-mediated dilation, hypertension, stroke, lower extremity arterial disease, major adverse cardiovascular events, myocardial infarction, peripheral artery disease, and all-cause mortality.

## Results

### Review and selection of primary studies

**Fig. 1:**
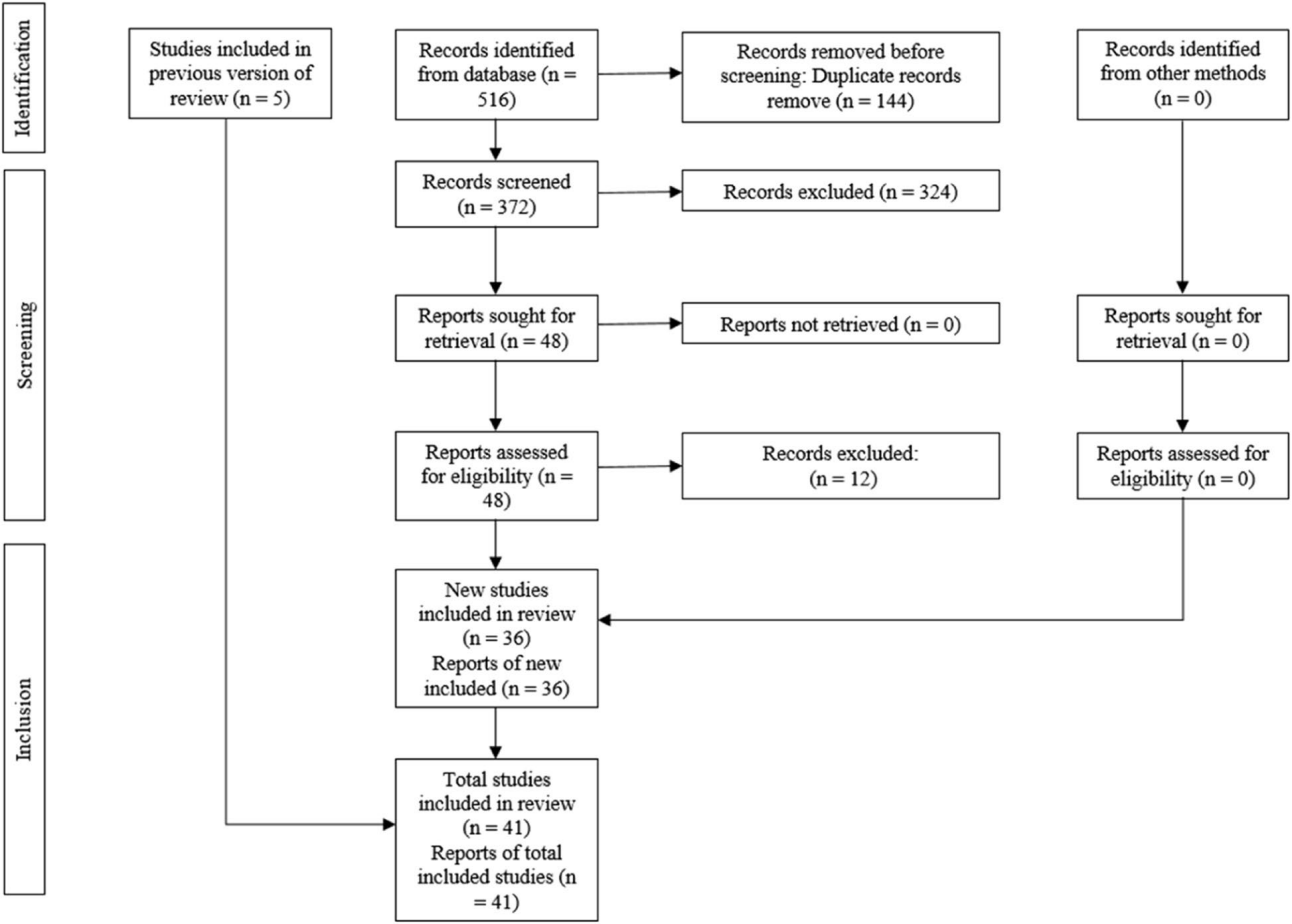
PRISMA flow diagram of the study selection process

The initial electronic database search yielded 516 articles, from which 372 remained after eliminating duplicates. In the first phase, the titles and abstracts of the identified studies were reviewed, resulting in 48 articles deemed eligible for full-text assessment. Ultimately, 36 SR remained from the initial search, and an additional 5 SR were included from previous studies, totaling 41 SR for qualitative synthesis. The exclusion criteria for articles are detailed in Table 2. The characteristics of the included studies are presented in Table 3. The entire process of study identification and selection is illustrated in Fig. 1.

**Table 2:** Reason for exclusion of studies

*Author(s)*	*Year*	*Reason for exclusion*
Ye et al. [27]	*2022*	CVD associated with PD treatment [27–37]
Liu et al. [28]	*2022*	
Navanar et al. [29]	*2022*	
Luo et al*. *[30]	*2021*	
Liu et al. [31]	*2019*	
Roca-Millan et al. [32]	*2018*	
Merchant et al. [33]	*2017*	
Liu et al*. *[34]	*2017*	
Teeuw et al. [35]	*2014*	
Li et al. [36]	*2014*	
Deng et al. [37]	*2013*	
Salhi et al. [38]	*2019*	Focused on comparisons with animal studies [38]

**Table 3:** Characteristics of included studies

**Authors**	**Year**	**Study Design**	**Country**	**Included Study Design**	**Number of Studies in the Qualitative Analysis**	**Number of Studies in the Quantitative Analysis**	**Outcomes**		**Conclusions**
Alwithanani et al. [39]	2023	SR and MA	Saudi Arabia	C	32	30	CVD	RR = 1.20	Individuals with PD consistently show a slightly elevated risk of CVD, particularly among men and those with severe PD.
							Stroke	RR = 1.24	
							CHD	RR = 1.14	
							MI	RR = 1.12	
							CVD – Men	RR = 1.16	
							CVD – Women	RR = 1.11	
							CVD – Mild PD	RR = 1.09	
							CVD – Moderate PD	RR = 1.23	
							CVD – Severe PD	RR = 1.25	
Guo et al. [40]	2023	SR and MA	China	C	42	39	MACE	RR = 1.24 (1.15 – 1.34)	PD is linked to an increased risk of MACE, CHD, MI, stroke, cardiac death, and all-cause mortality.
							CHD	RR = 1.20 (1.12 – 1.29)	
							MI	RR = 1.14 (1.06 – 1.22)	
							Stroke	RR = 1.26 (1.15 – 1.37)	
							Cardiac death	RR = 1.42 (1.10 – 1.84)	
							All-cause mortality	RR = 1.31 (1.29 – 1.38)	
Leelaviwat et al. [41]	2023	SR and MA	United State	C	4	3	AF / AFL	OR = 1.33 (1.29 – 1.38)	There is an association between PD and an elevated risk of AF/AFL.
Zhang et al. [42]	2023	SR	China	C, CC and CS	8	0	AF/AFL, MACE, cardiac arrhythmias and stroke	Patients with PD are at risk for AF/AFL, MACE, cardiac arrhythmias and stroke	PD is associated with AF/AFL, MACE, cardiac arrhythmias and stroke.
Leng et al. [43]	2023	SR and MA	China	C and CC	26	26	CVD	OR = 1.25 (1.13 – 1.38)	PD is correlated with a higher risk of CVD regardless of gender.
							CVD – Men	OR = 1.22 (1.12 – 1.34)	
							CVD – Women	OR = 1.11 (1.05 – 1.17)	
							CAD	OR = 1.16 (1.08 – 1.24)	
							CAD – Men	OR = 1.19 (1.09 – 1.30)	
							CAD – Women	OR = 1.18 (1.02 – 1.36)	
							Stroke	OR = 1.22 (1.08 – 1.37)	
							Stroke – Men	OR = 1.29 (1.08 – 1.37)	
							Stroke – Women	OR = 1.10 (1.09 – 1.11)	
Meregildo-Rodríguez et al. [44]	2022	SR and MA	Peru	C, CC and CS	46	46	ACS	OR = 1.35 (1.25 – 1.45)	PD is associated with a heightened risk of CVD irrespective of gender.
							ACS – North America	OR = 1.30 (1.16 – 1.46)	
							ACS – South America	OR = 4.43 (2.39 – 8.23)	
							ACS – Europe	OR = 1.92 (1.59 – 2.31)	
							ACS – Asia	OR = 1.09 (0.96 – 1.25)	
							ACS – Men	OR = 1.48 (1.11 – 1.97)	
							ACS – Women	OR = 1.96 (0.62 – 6.17)	
Xu et al. [45]	2022	SR and MA	China	C, CC and CS	28	21	HT	OR = 1.20 (1.10 – 1.30)	The relationship between tooth loss and HT may be bidirectional.
							HT –Age	OR = 1.40 (1.21 – 1.58)	
							HT – Men	OR = 1.22 (0.90 – 1.55)	
							HT – Women	OR = 1.20 (1.11 – 1.29)	
Leelapatana et al. [46]	2022	SR	Thailand	C and CC	5	0	AF / AFL	Periodontitis and the number of teeth lost were associated with AF/AFL.	Periodontitis and the number of teeth lost were associated with AF/AFL.
Tada et al. [47]	2022	SR and MA	Japan	C and CS	24	3	HT	OR = 2.22 (2.00 – 2.45)	Individuals with fewer remaining teeth or a greater extent of tooth loss tend to exhibit a higher prevalence of HT.
Qin et al. [48]	2021	SR and MA	China	C	10	10		OR = 1.13 (1.04 – 1.21)	PD is modestly associated with MI risk, especially in women.
							MI – Men	OR = 1.05 (0.89 – 1.24)	
							MI – Women	OR = 1.39 (1.17 – 1.65)	
Wang et al. [49]	2021	SR and MA	China	CS	12	10	CAC	OR = 4.42 (2.28 – 8.58)	PD is associated with CAC.
							CAC – Severe PD	OR = 6.40 (1.03 – 39.78)	
							CAC – Moderate PD	OR = 2.43 (1.04 – 5.70)	
Beukers et al. [50]	2021	SR and MA	Netherlands	C	75	44	ACVD	HR = 2.27 (1.50 – 3.43)	Having fewer teeth is identified as a risk factor for both ACVD and mortality.
							ACVD	RR = 2.93 (1.92 – 4.50)	
							All-cause mortality	HR = 2.47 (2.40 – 2.54)	
							All-cause mortality	RR = 2.27 (1.82 – 2.83)	
Larvin et al. [51]	2021	SR and MA	United Kingdom	C and RCT	32	30	CVD	RR = 1.20 (1.14 – 1.28)	There is a modest but consistently increased risk of CVD in PD populations, in men and people with severe PD.
							CVD – Men	RR = 1.16 (1.08 – 1.25)	
							CVD – Women	RR = 1.11 (1.02 – 1.22)	
							CVD – Mild PD	RR = 1.09 (1.05 – 1.14)	
							CVD – Moderate PD	RR = 1.23 (1.14 – 1.32)	
							CVD – Severe PD	RR = 1.25 (1.15 – 1.35)	
							CVD – Asia/Australia	RR = 1.20 (1.11 – 1.30)	
							CVD – Europe	RR = 1.36 (1.20 – 1.54)	
							CVD – North America	RR = 1.15 (1.09 – 1.22)	
							Stroke	RR = 1.24 (1.12 – 1.38)	
							CHD	RR = 1.14 (1.08 – 1.21)	
							MI	RR = 1.12 (0.96 – 1.30)	
Sun et al. [52]	2021	SR and MA	China	CC	18	18	CHD	OR = 3.42 (2.58 – 4.53)	PD might cause CHD susceptibility in the Chinese population.
Gao et al. [53]	2021	SR and MA	China	C	11	11	CHD	RR = 1.18 (1.10 – 1.26)	Periodontitis serves as a risk factor for CHD, and the number of extracted teeth is positively correlated with CHD risk.
							CHD	RR = 1.20 (1.12 – 1.27)	
Bodanese et al. [54]	2021	SR and MA	Brazil	C and CS	6	4	MI	RR = 2.62 (1.47 – 4.70)	Periodontitis is associated with MI.
Aguilera et al. [55]	2020	SR and MA	United Kingdom	C, CC, CS and CT	81	40	HT	OR = 1.22 (1.10 – 1.35)	PD is associated with increased odds of HT.
							HT – Severe PD	OR = 1.49 (1.09 – 2.05)	
Peng et al. [56]	2019	SR and MA	China	C and CC	18	18	All-cause mortality	RR = 1.57 (1.41 – 1.75)	Tooth loss, and in particular complete tooth loss (edentulism), could increase the risk of all-cause mortality.
							CVD mortality	RR = 1.83 (1.04 – 3.21)	
							CHD mortality	RR = 1.87 (1.01 – 3.47)	
Kumari et al. [57]	2019	SR	India	C, CC and CS	11	0	MI and CVD	There is an association between MI and CVD with PD	PD is mostly associated with MI and CVD.
Fagundes et al. [58]	2019	SR and MA	Brazil	C and CC	10	10	Stroke	RR = 2.31 (1.39 – 3.84)	Periodontitis may represent a risk factor for stroke, especially in ischemic events.
							Ischemic stroke	RR = 2.72 (2.00 – 3.71)	
Kaschwich et al. [60	2019	SR	Germany	C, CC and CS	10	0	PAOD	There is an association between PD and PAOD	The evidence presented supports an association between PD and PAOD.
Wang et al. [59]	2019	SR and MA	China	CC and CS	25	25	PAD	OR = 1.60 (1.41 – 1.82)	Periodontitis independently contributes to the increased incidence of both CaD and LEAD.
							LEAD	OR = 3.00 (2.23 – 4.04)	
							CaD	OR = 1.39 (1.24 – 1.56)	
Cheng et al. [60]	2018	SR and MA	China	C	17	17	CHD	RR = 1.52 (1.37 – 1.69)	Tooth loss was independently associated with deleterious CHD and stroke risk increment.
							CHD – Men	RR = 1.92 (1.34 – 2.50)	
							CHD – Women	RR = 1.48 (1.20 – 1.76)	
							CHD – Caucasia	RR = 1.55 (1.35 – 1.75)	
							CHD – Asia	RR = 1.38 (1.21 – 1.56)	
							Stroke	RR = 1.18 (1.11 – 1.25)	
							Stroke – Caucasia	RR = 1.25 (1.18 – 1.32)	
							Stroke – Asia	RR = 1.12 (1.01 – 1.23)	
Yang et al. [61]	2018	SR and MA	China	C, CC and CS	7	7	PAD	RR = 1.70 (1.25 – 2.29)	There is a significant relationship between periodontitis and PAD.
Xu et al. [62]	2017	SR and MA	China	C, CC and CS	20	20	MI	OR = 2.02 (1.59 – 2.57)	PD is associated with increased risk of future MI.
							MI – America	OR = 1.44 (1.16 – 1.78)	
							MI – Asia	OR = 2.93 (1.52 – 5.65)	
							MI – Europe	OR = 2.44 (1.01 – 5.86)	
							MI – Men	OR = 1.18 (0.96 – 1.44)	
							MI – Women	OR = 1.64 (1.20 – 2.25)	
Leira et al. [63]	2017	SR and MA	Spain	C and CC	8	8	Ischemic stroke	RR = 2.88 (1.53 – 5.41)	There is an association between periodontitis and ischemic stroke.
Shi et al. [64]	2016	SR and MA	China	CC	17	17	MI	OR = 2.53 (1.93 – 3.32)	There is a significant association between MI and periodontitis.
							MI – Europe	OR = 2.85 (1.95 – 4.14)	
							MI – United State	OR = 1.68 (1.18 – 2.39)	
							MI – Asia	OR = 8.79 (2.36 – 32.69)	
Zeng et al. [65]	2016	SR and MA	China	CC and CS	15	15	CA	OR = 1.27 (1.14 – 1.41)	The presence of PD was associated with CA.
							CA – Moderate PD	OR = 1.10 (1.04 – 1.16)	
							CA – Severe PD	OR = 1.14 (1.06 – 1.23)	
Martín-Cabezas et al. [66]	2016	SR and MA	France	C, CC and CS	25	18	HT	OR = 1.50 (1.27 – 1.78)	PDs are linked to a higher risk of HT, especially in cases of severe periodontitis.
							HT – Severe PD	OR = 1.40 (1.01 – 1.94)	
Schmitt et al. [67]	2015	SR and MA	France	C, CC, CS and RCT	10	7	AS – PWV	MD = 0.85 (0.53 – 1.16)	Patients with periodontitis tend to exhibit higher PWV values compared to control groups.
Lafon et al. [68]	2014	SR and MA	France	C	9	9	Stroke	RR = 1.63 (1.25 – 2.00)	Both periodontitis and tooth loss are associated with a higher likelihood of stroke occurrence.
							Ischemic + Hemorrhagic stroke	RR = 1.72 (1.20 – 2.25)	
							Ischemic stroke	RR = 1.53 (1.00 – 2.07)	
							Stroke	RR = 1.39 (1.13 – 1.65)	
							Ischemic + Hemorrhagic stroke	RR = 1.35 (1.05 – 1.66)	
							Ischemic stroke	RR = 1.50 (1.00 – 2.02)	
Orlandi et al. [69]	2014	SR and MA	United Kingdom	CC, CS, RCT and CT	35	22	c–IMT	MD = 0.08 (0.07 – 0.09)	There is an association between increased c-IMT, impaired FMD and PD.
							FMD	MD = -5.10 (-8.11 – -2.08)	
Dietrich et al. [70]	2013	SR	United Kingdom	C and CC	12	0	CHD, PAD, CvD and ACVD	There is a positive association between the various measures of PD and the incidence of ACVD, CvD, CHD and PAD; being stronger in younger adults	There is evidence for an increased risk of ACVD, CvD, CHD and PAD in patients with PD compared to patients without.
Polzer et al. [71]	2012	SR and MA	Germany	C	23	2	All-cause mortality	HR = 1.31 (1.03 – 1.65)	The number of teeth replaced affects circulatory mortality.
Sfyroeras et al. [72]	2012	SR and MA	Greece	C and CC	13	13	Stroke	OR = 2.63 (1.59 – 4.34)	Periodontitis is associated with increased risk of stroke.
							Stroke	RR = 1.48 (1.14 – 1.92)	
Blaizot et al. [73]	2009	SR and MA	France	C, CC and CS	32	29	CVD	OR = 2.35 (1.87 – 2.96)	Subjects with PDs have higher probabilities and higher risks of developing CVDs.
							CVD	RR = 1.34 (1.27 – 1.42)	
Humphrey et al. [74]	2008	SR and MA	United State	C	7	7	CHD	RR = 1.24 (1.01 – 1.51)	PD serves as either a risk factor or a marker for CHD.
							CHD – Men	RR = 1.23 (0.92 – 1.64)	
							CHD – Women	RR = 1.59 (1.28 – 1.96)	
							CHD	RR = 1.34 (1.10 – 1.63)	
Bahekar et al. [75]	2007	SR and MA	United State	C, CC and CS	15	15	CHD	RR = 1.14 (1.07 – 1.21)	PD may represent a risk factor for CHD.
							CHD	RR = 2.23 (1.59 – 3.12)	
							CHD	RR = 1.04 (0.85 – 1.28)	
Khader et al. [76]	2004	SR and MA	Jordan	C, CC and CS	11	11	CHD	RR = 1.15 (1.06 – 1.25)	PD increases the risk of CHD and CvD.
							CvD	RR = 1.17 (1.03 – 1.34)	
							CHD	RR = 1.09 (0.73 – 1.62)	
							CvD	RR = 1.46 (0.80 – 2.66)	
Janket et al. [77]	2003	SR and MA	United State	C	9	9	CVD	RR = 1.19 (1.10 – 1.40)	PD appears to be associated with an increase in risk of future CVD and stroke.
							Stroke	RR = 2.85 (1.78 – 4.56)	
Madianos et al. [78]	2002	SR	United State	C, CC and CS	21	0	CHD	There is a significant association between periodontitis and tooth loss with CHD	Periodontitis and tooth loss presents an increased risk of CHD.

*SR* systematic review, *MA* meta-analysis, *RCT* randomized controlled trial,*CT* clinical trial, *CS* cross-sectional, *C* cohort, *CC* case and control, *PD* periodontal disease, *PDs* periodontal diseases, *CVD* cardiovascular disease, *CVDs* cardiovascular diseases, *CvD* cerebrovascular disease, *ACVD* atherosclerotic cardiovascular disease, *HT* hypertension, *CHD* coronary heart disease, *MI* myocardial infarction, *MACE* major adverse cardiovascular events, *AF* atrial fibrillation, *AFL* atrial flutter, *CAD* coronary artery disease, *ACS* acute coronary syndrome, *CAC* carotid artery calcification, *PAOD* peripheral arterial occlusive disease, *PAD* peripheral artery disease, *LEAD* lower extremity arterial disease, *CaD* carotid artery disease, *CA* carotid atherosclerosis, *AS* arterial stiffness, *PWV* pulse wave velocity, *c–IMT* carotid intima–media thickness, *FMD* flow–mediated dilation, *OR* odds ratio, *RR* risk/rate ratio, *HR* hazard ratio

### Assessment of methodological quality and quality of evidence

Twenty six SR [42–47, 49–63, 66, 67, 69, 78, 79] were considered to have high confidence, six SR [40, 48, 64, 68, 73, 75] had low confidence, and nine SR [39, 41, 65, 70–72, 74, 76, 77] had critically low confidence (Table 4).

**Table 4:** Assessment of the methodological quality and the quality of the evidence of the included studies

Authors	Year	AMSTAR-2																Overall Confidence
		**1**	2 *	**3**	4 *	**5**	**6**	7 *	**8**	9 *	**10**	11 *	**12**	13 *	**14**	15 *	**16**	
Alwithanani et al. [39]	2023	Yes	No	Yes	Yes partial	Yes	Yes	No	Yes	Yes	Yes	Yes	Yes	Yes	Yes	Yes	Yes	Critically low
Guo et al. [40]	2023	Yes	No	Yes	Yes partial	Yes	Yes	Yes	Yes	Yes	Yes	Yes	Yes	Yes	Yes	Yes	Yes	Low
Leelaviwat et al. [41]	2023	Yes	No	Yes	Yes partial	No	No	Yes	Yes	No	Yes	Yes	No	No	Yes	Yes	Yes	Critically low
Zhang et al. [42]	2023	Yes	Yes	Yes	Yes partial	Yes	Yes	Yes	Yes	Yes	Yes	No meta-analysis		Yes	Yes	No meta-analysis	Yes	High
Leng et al. [43]	2023	Yes	Yes	Yes	Yes partial	Yes	Yes	Yes	Yes	Yes	Yes	Yes	Yes	Yes	Yes	Yes	Yes	High
Meregildo-Rodríguez et al. [44]	2022	Yes	Yes	Yes	Yes	Yes	Yes	Yes	Yes	Yes	Yes	Yes	Yes	Yes	Yes	Yes	Yes	High
Xu et al. [45]	2022	Yes	Yes	Yes	Yes	Yes	Yes	Yes	Yes	Yes	Yes	Yes	Yes	Yes	Yes	Yes	Yes	High
Leelapatana et al. [46]	2022	Yes	Yes	Yes	Yes	Yes	Yes	Yes	Yes	Yes	Yes	No meta-analysis		Yes	Yes	No meta-analysis	Yes	High
Tada et al. [47]	2022	Yes	Yes partial	Yes	Yes partial	Yes	Yes	Yes	Yes	Yes	Yes	Yes	Yes	Yes	Yes	Yes	Yes	High
Qin et al. [48]	2021	Yes	No	Yes	Yes partial	Yes	Yes	Yes	Yes	Yes	Yes	Yes	Yes	Yes	Yes	Yes	Yes	Low
Wang et al. [49]	2021	Yes	Yes	Yes	Yes partial	Yes	Yes	Yes	Yes	Yes	Yes	Yes	Yes	Yes	Yes	Yes	Yes	High
Beukers et al. [50]	2021	Yes	Yes partial	Yes	Yes partial	Yes	Yes	Yes	Yes	Yes	Yes	Yes	Yes	Yes	Yes	Yes	Yes	High
Larvin et al. [51]	2021	Yes	Yes	Yes	Yes	Yes	Yes	Yes	Yes	Yes	Yes	Yes	Yes	Yes	Yes	Yes	Yes	High
Sun et al. [52]	2021	Yes	Yes partial	Yes	Yes partial	Yes	Yes	Yes	Yes	Yes	Yes	Yes	Yes	Yes	Yes	Yes	Yes	High
Gao et al. [53]	2021	Yes	Yes partial	Yes	Yes partial	Yes	Yes	Yes	Yes	Yes	Yes	Yes	Yes	Yes	Yes	Yes	Yes	High
Bodanese et al. [54]	2021	Yes	Yes	Yes	Yes	Yes	Yes	Yes	Yes	Yes	Yes	Yes	Yes	Yes	Yes	Yes	Yes	High
Aguilera et al. [55]	2020	Yes	Yes	Yes	Yes	Yes	Yes	Yes	Yes	Yes	Yes	Yes	Yes	Yes	Yes	Yes	Yes	High
Peng et al. [56]	2019	Yes	Yes partial	Yes	Yes partial	Yes	Yes	Yes	Yes	Yes	Yes	Yes	Yes	Yes	Yes	Yes	Yes	High
Kumari et al.[57]	2019	Yes	Yes partial	Yes	Yes	Yes	Yes	Yes partial	Yes	Yes partial	Yes	No meta-analysis		Yes	Yes	No meta-analysis	Yes	High
Fagundes et al. [58]	2019	Yes	Yes	Yes	Yes	Yes	Yes	Yes	Yes	Yes partial	Yes	Yes	Yes	Yes	Yes	Yes	Yes	High
Kaschwich et al. [79]	2019	Yes	Yes partial	Yes	Yes	Yes	Yes	Yes	Yes	Yes	Yes	No meta-analysis		Yes	Yes	No meta-analysis	Yes	High
Wang et al. [59]	2019	Yes	Yes partial	Yes	Yes	Yes	Yes	Yes	Yes	Yes	Yes	Yes	Yes	Yes	Yes	Yes	Yes	High
Cheng et al. [60]	2018	Yes	Yes	Yes	Yes partial	Yes	Yes	Yes	Yes	Yes	Yes	Yes	Yes	Yes	Yes	Yes	Yes	High
Yang et al. [61]	2018	Yes	Yes partial	Yes	Yes partial	Yes	Yes	Yes	Yes	Yes	Yes	Yes	Yes	Yes	Yes	Yes	Yes	High
Xu et al. [62]	2017	Yes	Yes partial	Yes	Yes partial	Yes	Yes	Yes	Yes	Yes	Yes	Yes	Yes	Yes	Yes	Yes	Yes	High
Leira et al. [63]	2017	Yes	Yes	Yes	Yes	Yes	Yes	Yes	Yes	Yes partial	Yes	Yes	Yes	Yes	Yes	Yes	Yes	High
Shi et al. [64]	2016	Yes	No	Yes	Yes partial	Yes	Yes	Yes	Yes	Yes	Yes	Yes	Yes	Yes	Yes	Yes	Yes	Low
Zeng et al. [65]	2016	Yes	Yes partial	Yes	Yes partial	Yes	Yes	Yes	Yes	No	Yes	Yes	No	No	Yes	Yes	Yes	Critically low
Martín-Cabezas et al. [66]	2016	Yes	Yes partial	Yes	Yes partial	Yes	Yes	Yes partial	Yes	Yes partial	Yes	Yes	Yes	Yes	Yes	Yes	Yes	High
Schmitt et al. [67]	2015	Yes	Yes partial	Yes	Yes	Yes	Yes	Yes	Yes	Yes	Yes	Yes	Yes	Yes	Yes	Yes	Yes	High
Lafon et al. [68]	2014	Yes	No	Yes	Yes partial	Yes	Yes	Yes	Yes	Yes partial	Yes	Yes	Yes	Yes	Yes	Yes	Yes	Low
Orlandi et al. [69]	2014	Yes	Yes partial	Yes	Yes	Yes	Yes	Yes partial	Yes	Yes	Yes	Yes	Yes	Yes	Yes	Yes	Yes	High
Dietrich et al. [70]	2013	Yes	No	Yes	No	Yes	Yes	No	Yes	No	Yes	No meta-analysis		No	yes	No meta-analysis	Yes	Critically low
Polzer et al. [71]	2012	Yes	No	Yes	No	Yes	Yes	Yes	Yes	Yes partial	Yes	Yes	Yes	Yes	Yes	Yes	Yes	Critically low
Sfyroeras et al. [72]	2012	Yes	No	Yes	No	Yes	Yes	No	Yes	No	Yes	Yes	No	No	Yes	Yes	Yes	Critically low
Blaizot et al. [73]	2009	Yes	No	Yes	Yes partial	Yes	Yes	Yes	Yes	Yes partial	Yes	Yes	Yes	Yes	Yes	Yes	Yes	Low
Humphrey et al. [74]	2008	Yes	No	Yes	No	Yes	Yes	Yes	Yes	Yes partial	Yes	Yes	Yes	Yes	Yes	Yes	Yes	Critically low
Bahekar et al. [75]	2007	Yes	No	Yes	Yes partial	Yes	Yes	Yes partial	Yes	Yes partial	Yes	Yes	Yes	Yes	Yes	Yes	Yes	Low
Khader et al. [76]	2004	Yes	No	Yes	No	Yes	Yes	Yes partial	Yes	No	Yes	Yes	No	No	Yes	Yes	Yes	Critically low
Janket et al. [77]	2003	Yes	No	Yes	No	Yes	Yes	Yes	Yes	Yes partial	Yes	Yes	Yes	Yes	Yes	Yes	Yes	Critically low
Madianos et al. [78]	2002	Yes	Yes partial	Yes	Yes	Yes	Yes	Yes	Yes	Yes partial	Yes	No meta-analysis		Yes	yes	No meta-analysis	Yes	High

*AMSTAR *A MeaSurement Tool to Assess Systemic Reviews

1 = Did the research questions and inclusion criteria for the review include the components of PICO? 2 = Did the report of the review contain an explicit statement that the review methods were established prior to the conduct of the review and did the report justify any significant deviations from the protocol? 3 = Did the review authors explain their selection of the study designs for inclusion in the review? 4 = Did the review authors use a comprehensive literature search strategy? 5 = Did the review authors perform study selection in duplicate? 6 = Did the review authors perform data extraction in duplicate? 7 = Did the review authors provide a list of excluded studies and justify the exclusions? 8 = Did the review authors describe the included studies in adequate detail? 9 = Did the review authors use a satisfactory technique for assessing the risk of bias (RoB) in individual studies that were included in the review? 10 = Did the review authors report on the sources of funding for the studies included in the review? 11 = If meta-analysis was performed, did the review authors use appropriate methods for statistical combination of results? 12 = If meta-analysis was performed, did the review authors assess the potential impact of RoB in individual studies on the results of the meta-analysis or other evidence synthesis? 13 = Did the review authors account for RoB in primary studies when interpreting/discussing the results of the review? 14 = Did the review authors provide a satisfactory explanation for, and discussion of, any heterogeneity observed in the results of the review? 15 = If they performed quantitative synthesis, did the review authors carry out an adequate investigation of publication bias (small study bias) and discuss its likely impact on the results of the review? 16 = Did the review authors report any potential sources of conflict of interest, including any funding they received for conducting the review?

^*^Critical domain

### Overlapping

A total of 839 primary studies were identified within the SR. Of these, approximately 74% of the primary studies overlapped across multiple SR. Eighty-one studies were duplicated in two reviews, while forty-three appeared in three reviews. Additionally, eleven studies were found in four reviews, with seven studies overlapping in five reviews. Similarly, eight studies were included in six reviews, seven in seven reviews, and five in eight reviews. Furthermore, three studies were featured in nine reviews, one in ten reviews, another in eleven reviews, two in twelve reviews, one in fourteen reviews, one in fifteen reviews, and one in sixteen reviews. Further details on the overlap and characteristics of the primary studies can be found in Mat. supl.1.

### Synthesis of results

The summaries of the findings are displayed in Table 5.

**Table 5:** Synthesis of the results of the included studies

**Authors**	**Outcome**		**Condition**	**Association**	
Alwithanani et al.	CVD	RR = 1.20	PD	Yes	
	Stroke	RR = 1.24		Yes	
	CHD	RR = 1.14		Yes	
	MI	RR = 1.12		Yes	
	CVD – Men	RR = 1.16		Yes	
	CVD – Women	RR = 1.11		Yes	
	CVD – Mild PD	RR= 1.09		Yes	
	CVD – Moderate PD	RR = 1.23		Yes	
	CVD – Severe PD	RR = 1.25		Yes	
Guo et al.	MACE	RR = 1.24 (1.15 – 1.34)	PD	Yes	
	CHD	RR = 1.20 (1.12 – 1.29)		Yes	
	MI	RR = 1.14 (1.06 – 1.22)		Yes	
	Stroke	RR = 1.26 (1.15 – 1.37)		Yes	
	Cardiac death	RR = 1.42 (1.10 – 1.84)		Yes	
	All-cause mortality	RR = 1.31 (1.07 – 1.61)		Yes	
Leelaviwat et al.	AF / AFL	OR = 1.33 (1.29 – 1.38)	PD	Yes	
Zhang et al.	AF/AFL, MACE, cardiac arrhythmias and stroke.	Patients with PD are at risk for AF/AFL, MACE, cardiac arrhythmias and stroke.	PD	Yes	
Leng et al.	CVD	OR = 1.25 (1.13 – 1.38)	PD	Yes	
	CVD – Men	OR = 1.22 (1.12 – 1.34)		Yes	
	CVD – Women	OR = 1.11 (1.05 – 1.17)		Yes	
	CAD	OR = 1.16 (1.08 – 1.24)		Yes	
	CAD – Men	OR = 1.19 (1.09 – 1.30)		Yes	
	CAD – Women	OR = 1.18 (1.02 – 1.36)		Yes	
	Stroke	OR = 1.22 (1.08 – 1.37)		Yes	
	Stroke – Men	OR = 1.29 (1.08 – 1.37)		Yes	
	Stroke – Women	OR = 1.10 (1.09 – 1.11)		Yes	
Meregildo-Rodríguez et al	ACS	OR = 1.35 (1.25 – 1.45)		Yes	
	ACS – North America	OR = 1.30 (1.16 – 1.46)		Yes	
	ACS – South America	OR = 4.43 (2.39 – 8.23)		Yes	
	ACS – Europe	OR = 1.92 (1.59 – 2.31)		Yes	
	ACS – Asia	OR = 1.09 (0.96 – 1.25)		No	
	ACS – Men	OR = 1.48 (1.11 – 1.97)		Yes	
	ACS – Women	OR = 1.96 (0.62 – 6.17)		Yes	
Xu et al.	HT	OR = 1.20 (1.10 – 1.30)	Tooth loss	Yes	
	HT – Age	OR = 1.40 (1.21 – 1.58)		Yes	
	HT – Men	OR = 1.22 (0.90 – 1.55)		No	
	HT – Women	OR = 1.20 (1.11 – 1.29)		Yes	
Leelapatana et al.	AF/AFL	Periodontitis and the number of teeth lost were associated with AF/AFL.	PD and Tooth loss	Yes	
Tada et al.	HT	OR = 2.22 (2.00 – 2.45)	Tooth loss	Yes	
Qin et al.	MI	OR = 1.13 (1.04 – 1.21)	PD	Yes	
	MI – Men	OR = 1.05 (0.89 – 1.24)		No	
	MI – Women	OR = 1.39 (1.17 – 1.65)		Yes	
Wang et al.	CAC	OR = 4.42 (2.28 – 8.58)	PD	Yes	
	CAC – Severe PD	OR = 6.40 (1.03 – 39.78)		Yes	
	CAC – Moderate PD	OR = 2.43 (1.04 – 5.70)		Yes	
Beukers et al.	ACVD	HR = 2.27 (1.50 – 3.43)	Tooth loss	Yes	
	ACVD	RR = 2.93 (1.92 – 4.50)		Yes	
	All-cause mortality	HR = 2.47 (2.40 – 2.54)		Yes	
	All-cause mortality	RR = 2.27 (1.82 – 2.83)		Yes	
Larvin et al.	CVD	RR = 1.20 (1.14 – 1.28)	PD	Yes	
	CVD – Men	RR = 1.16 (1.08 – 1.25)		Yes	
	CVD – Women	RR = 1.11 (1.02 – 1.22)		Yes	
	CVD – Mild PD	RR = 1.09 (1.05 – 1.14)		Yes	
	CVD – Moderate PD	RR = 1.23 (1.14 – 1.32)		Yes	
	CVD – Severe PD	RR = 1.25 (1.15 – 1.35)		Yes	
	CVD – Asia/Australia	RR = 1.20 (1.11 – 1.30)		Yes	
	CVD – Europe	RR = 1.36 (1.20 – 1.54)		Yes	
	CVD – North America	RR = 1.15 (1.09 – 1.22)		Yes	
	Stroke	RR = 1.24 (1.12 – 1.38)		Yes	
	CHD	RR = 1.14 (1.08 – 1.21)		Yes	
	MI	RR = 1.12 (0.96 – 1.30)		No	
Sun et al.	CHD	OR = 3.42 (2.58 – 4.53)	PD	Yes	
Gao et al.	CHD	RR = 1.18 (1.10 – 1.26)	PD	Yes	
	CHD	RR = 1.20 (1.12 – 1.27)	Tooth loss	Yes	
Bodanese et al.	MI	RR = 2.62 (1.47 – 4.70)	PD	Yes	
Aguilera et al.	HT	OR = 1.22 (1.10 – 1.35)	PD	Yes	
	HT – Severe PD	OR = 1.49 (1.09 – 2.05)		Yes	
Peng et al.	All-cause mortality	RR = 1.57 (1.41 – 1.75)	Tooth loss	Yes	
	CVD mortality	RR = 1.83 (1.04 – 3.21)		Yes	
	CHD mortality	RR = 1.87 (1.01 – 3.47)		Yes	
Kumari et al.	MI and CVD	There is an association between MI and CVD with PD	PD	Yes	
Fagundes et al.	Stroke	RR = 2.31 (1.39 – 3.84)	PD	Yes	
	Ischemic stroke	RR = 2.72 (2.00 – 3.71)		Yes	
Kaschwich et al.	PAOD	There is an association between PD and PAOD	PD	Yes	
Wang et al.	PAD	OR = 1.60 (1.41 – 1.82)	PD	Yes	
	LEAD	OR = 3.00 (2.23 – 4.04)		Yes	
	CaD	OR = 1.39 (1.24 – 1.56)		Yes	
Cheng et al.	CHD	RR = 1.52 (1.37 – 1.69)	Tooth loss	Yes	
	CHD – Men	RR = 1.92 (1.34 – 2.50)		Yes	
	CHD – Women	RR = 1.48 (1.20 – 1.76)		Yes	
	CHD – Caucasia	RR = 1.55 (1.35 – 1.75)		Yes	
	CHD – Asia	RR = 1.38 (1.21 – 1.56)		Yes	
	Stroke	RR = 1.18 (1.11 – 1.25)		Yes	
	Stroke – Caucasia	RR = 1.25 (1.18 – 1.32)		Yes	
	Stroke – Asia	RR = 1.12 (1.01 – 1.23)		Yes	
Yang et al.	PAD	RR = 1.70 (1.25 – 2.29)	PD	Yes	
Xu et al.	MI	OR = 2.02 (1.59 – 2.57)	PD	Yes	
	MI – America	OR = 1.44 (1.16 – 1.78)		Yes	
	MI – Asia	OR = 2.93 (1.52 – 5.65)		Yes	
	MI – Europe	OR = 2.44 (1.01 – 5.86)		Yes	
	MI – Men	OR = 1.18 (0.96 – 1.44)		No	
	MI – Women	OR = 1.64 (1.20 – 2.25)		Yes	
Leira et al.	Ischemic stroke	RR = 2.88 (1.53 – 5.41)	PD	Yes	
Shi et al.	MI	OR = 2.53 (1.93 – 3.32)	PD	Yes	
	MI – Europe	OR = 2.85 (1.95 – 4.14)		Yes	
	MI – United State	OR = 1.68 (1.18 – 2.39)		Yes	
	MI – Asia	OR = 8.79 (2.36 – 32.69)		Yes	
Zeng et al.	CA	OR = 1.27 (1.14 – 1.41)	PD	Yes	
	CA – Moderate PD	OR = 1.10 (1.04 – 1.16)		Yes	
	CA – Severe PD	OR = 1.14 (1.06 – 1.23)		Yes	
Martín-Cabezas et al.	HT	OR = 1.50 (1.27 – 1.78)	PD	Yes	
	HT – Severe PD	OR = 1.40 (1.01 – 1.94)		Yes	
Schmitt et al.	AS – PWV	MD = 0.85 (0.53 – 1.16)	PD	Yes	
Lafon et al.	Stroke	RR = 1.63 (1.25 – 2.00)	PD	Yes	
	Ischemic + Hemorrhagic stroke	RR = 1.72 (1.20 – 2.25)		Yes	
	Ischemic stroke	RR = 1.53 (1.00 – 2.07)		Yes	
	Stroke	RR = 1.39 (1.13 – 1.65)	Tooth loss	Yes	
	Ischemic + Hemorrhagic stroke	RR = 1.35 (1.05 – 1.66)		Yes	
	Ischemic stroke	RR = 1.50 (1.00 – 2.02)		Yes	
Orlandi et al.	c-IMT	MD = 0.08 (0.07 – 0.09)	PD	Yes	
	FMD	MD = -5.10 (-8.11 – -2.08)		Yes	
Dietrich et al.	CHD, PAD, CvD and ACVD	There is a positive association between the various measures of PD and the incidence of ACVD, CvD, CHD and PAD; being stronger in younger adults.	PD	Yes	
Polzer et al.	All-cause mortality	HR = 1.31 (1.03 – 1.65)	Tooth loss	Yes	
Sfyroeras et al.	Stroke	OR = 2.63 (1.59 – 4.34)	PD	Yes	
	Stroke	RR = 1.48 (1.14 – 1.92)		Yes	
Blaizot et al.	CVD	OR = 2.35 (1.87 – 2.96)	PD	Yes	
	CVD	RR = 1.34 (1.27 – 1.42)		Yes	
Humphrey et al.	CHD	RR = 1.24 (1.01 – 1.51)	PD	Yes	
	CHD – Men	RR = 1.23 (0.92 – 1.64)		No	
	CHD – Women	RR = 1.59 (1.28 – 1.96)		Yes	
	CHD	RR = 1.34 (1.10 – 1.63)	Tooth loss	Yes	
Bahekar et al.	CHD	RR = 1.14 (1.07 – 1.21)	PD	Yes	
	CHD	OR = 2.23 (1.59 – 3.12)		Yes	
	CHD	RR = 1.04 (0.85 – 1.28)	Tooth loss	No	
Khader et al.	CHD	RR = 1.15 (1.06 – 1.25)	PD	Yes	
	CvD	RR = 1.17 (1.03 – 1.34)		Yes	
	CHD	RR = 1.09 (0.73 – 1.62)	Tooth loss	No	
	CvD	RR = 1.46 (0.80 – 2.66)		No	
Janket et al.	CVD	RR = 1.19 (1.10 – 1.40)	PD	Yes	
	Stroke	RR = 2.85 (1.78 – 4.56)		Yes	
Madianos et al.	CHD	There is a significant association between periodontitis and tooth loss with CHD	PD and Tooth loss	Yes	

*PD* periodontal disease, *CVD* cardiovascular disease, *CvD* cerebrovascular disease, *ACVD* atherosclerotic cardiovascular disease, *HT* hypertension, *CHD* coronary heart disease, *MI* myocardial infarction, *MACE* major adverse cardiovascular events, *AF* atrial fibrillation, *AFL* atrial flutter, *CAD* coronary artery disease, *ACS* acute coronary syndrome, *CAC* carotid artery calcification, *PAOD* peripheral arterial occlusive disease, *PAD* peripheral artery disease, *LEAD* lower extremity arterial disease, *CaD* carotid artery disease, *CA* carotid atherosclerosis, *AS* arterial stiffness, *PWV* pulse wave velocity, *c–IMT* carotid intima–media thickness, *FMD* flow–mediated dilation, *OR* odds ratio, *RR* risk/rate ratio, *HR* hazard ratio

### Cardiovascular disease (CVD)

Six SR [39, 43, 51, 57, 73, 77] included reported that there was an association between PD and CVD. Five SR [39, 43, 51, 73, 77] meta-analyzed the results and found that the OR ranged from 1.25 (CI: 1.13 to 1.38) [43] to 2.35 (CI: 1.87 to 2.96) [73] and the RR ranged from 1.19 (CI: 1.10 to 1.40) [77] to 1.20 (CI: 1.14 to 1.28) [51]. Kumari et al. [57] reported that there is an association between CVD and PD.

One SR [51] included reported that there was an association between PD and CVD for country or continent. This study meta-analyzed its results and found that the RR for Asia/Australia was 1.20 (CI: 1.11 to 1.30), for Europe was 1.36 (CI: 1.20 to 1.54), and for North America was 1.15 (CI: 1.09 to 1.22).

Three SR [39, 43, 51] included reported that there was an association between PD and CVD for sex. This studies meta-analyzed its results and found that the OR for men was 1.22 (CI: 1.12 to 1.34) [43] and for women was 1.11 (CI: 1.05 to 1.17) [43]; the RR for men was 1.16 (CI: 1.08 to 1.25) [51] and for women was 1.11 (CI: 1.02 to 1.22) [51].

Two SR [39, 51] included reported that there was an association between PD and CVD for severity PD. This studies meta-analyzed its results and found that the RR for mild PD was 1.09 (CI: 1.05 to 1.14) [51], for moderate PD was 1.23 (CI: 1.14 to 1.32) [51] and for severe PD was 1.25 (CI: 1.15 to 1.35) [51].

One SR [56] included reported that there was an association between tooth loss and CVD mortality. This study meta-analyzed its results and found that the RR was 1.83 (CI: 1.04 to 3.21).

### Cerebrovascular disease (CvD)

Two SR [70, 76] included reported that there was an association between PD and CvD, but there is no association with tooth loss. One SR [76] meta-analyzed its results and found that the RR for the PD was 1.17 (CI: 1.03 to 1.34), and for tooth loss was 1.46 (CI: 0.80 to 2.66). Dietrich et al. [70] reported that there is an association between CvD and PD.

### Atherosclerotic cardiovascular disease (ACVD)

Two SR [50, 70] included reported that there was an association between PD and tooth loss, with ACVD. One SR [50] meta-analyzed its results and found that the HR for the tooth loss was 2.27 (CI: 1.50 to 3.43) and RR was 2.93 (CI: 1.92 to 4.50). Dietrich et al. [70] reported that there is an association between ACVD and PD.

### Acute coronary syndrome (ACS)

One SR [44] included reported that there was an association between PD and ACS. This study meta-analyzed its results and found that the OR was 1.35 (CI: 1.25 to 1.45). The OR for North America was 1.30 (CI: 1.16 to 1.46), for South America was 4.43 (CI: 2.39 to 8.23), for Europe was 1.92 (CI: 1.59 to 2.31) and for Asia was 1.09 (CI: 0.96 to 1.25). The OR for men was 1.48 (CI: 1.11 to 1.97) and for women was 1.96 (CI: 0.62 to 6.17).

### Atrial fibrillation / Atrial flutter (AF/AFL)

Three SR [41, 42, 46] included reported that there was an association between PD and tooth loss, with AF/AFL. One SR [41] meta-analyzed its results and found that the OR was 1.33 (CI: 1.29 to 1.38). Zhang et al. [42] and Leelapatana et al. [46] reported that there is an association between AF/AFL and PD.

#### Arterial stiffness (AS)

One SR [67] included reported that there was an association between PD and AS. This study meta-analyzed its results and found that the MD was 0.85 (CI: 0.53 to 1.16).

#### Cardiac arrhythmias

One SR [42] included reported that there was an association between PD and cardiac arrhythmias.

#### Carotid atherosclerosis (CA)

One SR [65] included reported that there was an association between PD and CA. This study meta-analyzed its results and found that the OR was 1.27 (CI: 1.14 to 1.41). The OR for moderate PD was 1.10 (CI: 1.04 to 1.16) and for severe PD was 1.14 (CI: 1.06 to 1.23).

#### Carotid artery calcification (CAC)

One SR [49] included reported that there was an association between PD and CAC. This study meta-analyzed its results and found that the OR was 4.42 (CI: 2.28 to 8.58). The OR for moderate PD was 2.43 (CI: 1.04 to 5.70) and for severe PD was 6.40 (CI: 1.03 to 39.78).

#### Coronary artery disease (CAD)

One SR [43] included reported that there was an association between PD and CAD. This study meta-analyzed its results and found that the OR was 1.16 (CI: 1.08 to 1.24). The OR for men was 1.19 (CI: 1.09 to 1.30) and for women was 1.18 (CI: 1.02 to 1.36).

#### Carotid artery disease (CaD)

One SR [59] included reported that there was an association between PD and CaD. This study meta-analyzed its results and found that the OR was 1.39 (CI: 1.24 to 1.56).

#### Cardiac death

One SR [40] included reported that there was an association between PD and cardiac death. This study meta-analyzed its results and found that the RR was 1.42 (CI: 1.10 to 1.84).

#### Coronary heart disease (CHD)

Ten SR [39, 40, 51–53, 70, 74–76, 78] included reported that there was an association between PD and CHD. Eight SR [39, 40, 51–53, 74–76] meta-analyzed the results and found that the OR ranged from 2.23 (CI: 1.59 to 3.12) [75] to 3.42 (CI: 2.58 to 4.53) [52]; and the RR ranged from 1.14 (CI: 1.07 to 1.21) [75] to 1.24 (CI: 1.01 to 1.51) [74]. Dietrich et al. [70] and Madianos et al. [78] reported that there is an association between CHD and PD.

Four SR [53, 60, 74, 78] included reported that there was an association between tooth loss and CHD, but in two SR [75, 76] this association was not found. Five SR [53, 60, 74–76] meta-analyzed the results and found that the RR ranged from 1.04 (CI: 0.85 to 1.28) [75] to 1.52 (CI: 1.37 to 1.69) [60]. Madianos et al. [78] reported that there is an association between CHD and tooth loss.

One SR [60] included reported that there was an association between tooth loss and CHD for continent. This study meta-analyzed its results and found that the RR for Asia was 1.38 (CI: 1.21 to 1.56) and for Caucasia was 1.55 (CI: 1.35 to 1.75).

One SR [74] included reported that there was an association between PD and CHD for women. This study meta-analyzed its results and found that the RR for men was 1.23 (CI: 0.92 to 1.64) and for women was 1.59 (CI: 1.28 to 1.96).

One SR [60] included reported that there was an association between tooth loss and CHD for sex. This study meta-analyzed its results and found that the RR for men was 1.92 (CI: 1.34 to 2.50) and for women was 1.48 (CI: 1.20 to 1.76).

One SR [56] included reported that there was an association between tooth loss and CHD mortality. This study meta-analyzed its results and found that the RR was 1.87 (CI: 1.01 to 3.47).

#### Carotid intima – media thickness / Flow – mediated dilation (c–IMT/FMD)

One SR [69] included reported that there was an association between PD and c–IMT/FMD. This study meta-analyzed its results and found that the MD for c–IMT was 0.08 (CI: 0.07 to 0.09) and for FMD was –5.10 (CI: –8.11 to –2.08).

#### Hypertension (HT)

Four SR [45, 47, 55, 66] included reported that there was an association between PD and tooth loss, with HT. Two SR [55, 66] meta-analyzed its results and found that the OR for the PD ranged from 1.22 (CI: 1.10 to 1.35) [55] to 1.50 (CI: 1.27 to 1.78) [66] and for the tooth loss ranged from 1.20 (CI: 1.10 to 1.30) [45] to 2.22 (CI: 2.00 to 2.45) [47].

One SR [45] included reported that there was an association between tooth loss and HT for age. This study meta-analyzed its results and found that the OR was 1.40 (CI: 1.21 to 1.58).

One SR [45] included reported that there was an association between tooth loss and HT for women. This study meta-analyzed its results and found that the OR for men was 1.22 (CI: 0.90 to 1.55) and for women was 1.20 (CI: 1.11 to 1.29).

Two SR [55, 66] included reported that there was an association between PD and HT for severe PD. This studies meta-analyzed its results and found that the OR ranged from 1.40 (CI: 1.01 to 1.94) [66] to 1.49 (CI: 1.09 to 2.05) [55].

#### Stroke

Ten SR [39, 40, 42, 43, 51, 58, 60, 68, 72, 77] included reported that there was an association between PD and tooth loss, with stroke. Nine SR [39, 40, 43, 51, 58, 60, 68, 72, 77] meta-analyzed its results and found that the OR for the PD ranged from 1.22 (CI: 1.08 to 1.37) [43] to 2.63 (CI: 1.59 to 4.34) [72] and the RR ranged from 1.24 (CI: 1.12 to 1.38) [51] to 2.85 (CI: 1.78 to 4.56) [77]; and the RR for the tooth loss ranged from 1.18 (CI: 1.11 to 1.25) [60] to 1.39 (CI: 1.13 to 1.65) [68]. Zhang et al. [42] reported that there is an association between stroke and PD.

Three SR [58, 63, 68] included reported that there was an association between PD and tooth loss, with ischemic or hemorrhagic stroke. This studies meta-analyzed its results and found that the RR for PD ranged from 1.53 (CI: 1.00 to 2.07) [68] to 2.88 (CI: 1.53 to 5.41) [63] and for tooth loss ranged from 1.35 (CI: 1.05 to 1.66) [68] to 1.50 (CI: 1.00 to 2.02) [68].

One SR [60] included reported that there was an association between tooth loss and stroke for continent. This study meta-analyzed its results and found that the RR for Asia was 1.12 (CI: 1.01 to 1.23) and for Caucasia was 1.25 (CI: 1.18 to 1.32).

One SR [43] included reported that there was an association between PD and stroke for sex. This study meta-analyzed its results and found that the OR for men was 1.29 (CI: 1.08 to 1.37) and for women was 1.10 (CI: 1.09 to 1.11).

#### Lower extremity arterial disease (LEAD)

One SR [59] included reported that there was an association between PD and LEAD. This study meta-analyzed its results and found that the OR was 3.00 (CI: 2.23 to 4.04).

#### Major adverse cardiovascular event (MACE)

Two SR [40, 42] included reported that there was an association between PD and MACE. One SR [40] meta-analyzed its results and found that the RR was 1.24 (CI: 1.15 to 1.34). Zhang et al. [42] reported that there is an association between MACE and PD.

#### Myocardial infarction (MI)

Seven SR [39, 40, 48, 54, 57, 62, 64] included reported that there was an association between PD and MI, but in one SR [51] this association was not found. Seven SR [39, 40, 48, 51, 54, 62, 64] meta-analyzed its results and found that the OR ranged from 1.13 (CI: 1.04 to 1.21) [48] to 2.53 (CI: 1.93 to 3.32) [64], and the RR ranged from 1.12 (CI: 0.96 to 1.30) [51] to 2.62 (CI: 1.47 to 4.70) [54]. Kumari et al. [57] reported that there is an association between MI and PD.

Two SR [62, 64] included reported that there was an association between PD and MI for country or continent. This studies meta-analyzed its results and found that the OR for America was 1.44 (CI: 1.16 to 1.78) [62], for Asia ranged from 2.93 (CI: 1.52 to 5.65) [62] to 8.79 (CI: 2.36 to 32.69) [64], for Europe ranged from 2.44 (CI: 1.01 to 5.86) [62] to 2.85 (CI: 1.95 to 4.14) [64] and for United State was 1.68 (CI: 1.18 to 2.39) [64].

Two SR [48, 62] included reported that there was an association between PD and MI for women. This studies meta-analyzed its results and found that the OR for men ranged from 1.05 (CI: 0.89 to 1.24) [48] to 1.18 (CI: 0.96 to 1.44) [62], and for women ranged from 1.39 (CI: 1.17 to 1.65) [48] to 1.64 (CI: 1.20 to 2.25) [62].

#### Peripheral artery disease (PAD)

Three SR [59, 61, 70] included reported that there was an association between PD and PAD. Two SR [59, 61] meta-analyzed its results and found that the OR ranged from 1.60 (CI: 1.41 to 1.82) [59] to 1.70 (CI: 1.25 to 2.29) [61]. Dietrich et al. [70] reported that there is an association between PAD and PD.

#### Peripheral arterial occlusive disease (PAOD)

One SR [79] included reported that there was an association between PD and PAOD.

#### All-cause mortality

Four SR [40, 50, 56, 71] included reported that there was an association between PD and tooth loss, with all-cause mortality. This studies meta-analyzed its results and found that the RR for the PD was 1.31 (CI: 1.07 to 1.61) [40] and for tooth loss ranged from 1.57 (CI: 1.41 to 1.75) [56] to 2.27 (CI: 1.82 to 2.83) [50]; and the HR for the tooth loss ranged from 1.31 (CI: 1.03 to 1.65) [71] to 2.47 (CI: 2.40 to 2.54) [50].

## Discussion

In recent years, there has been an interest increase in examining and understanding the connection between PD and CVD. A considerable amount of research has been dedicated to exploring this topic, and the findings obtained support the existence of this association.

Currently, CVD has been the leading cause of global mortality for decades, impacting people of all races and ethnicities around the world [80]. Despite their significant prevalence, estimates provided by the World Health Organization (WHO) suggest that more than 75% of CVDs are preventable or treatable with appropriate resources [81].

The results of this review are consistent with the existing literature, which suggests a significant association between PD and CVD. Recent studies have shown that the chronic inflammation associated with PD may contribute to atherosclerosis and other cardiovascular conditions through mechanisms such as the dissemination of periodontal bacteria into the systemic circulation and the induction of a systemic inflammatory response (Hajishengallis 2012, Kinane 2017) [4, 8]. This finding underscores the importance of considering periodontal health as an integral component in the prevention of CVD.

For years, oral health researchers have explored the possible connection between CVD and PD, demonstrating that the mechanisms underlying this association include the chronic entry of periodontal bacteria into the vascular system (bacteremia), which triggers systemic inflammatory responses. and increased levels of systemic inflammation due to periodontitis. Furthermore, periodontitis and CVD share several genetic and environmental risk factors, such as smoking [82].

A similar study conducted by Madianos et al. [78] reported a significant association between tooth loss, an indicator of advanced PD, and the risk of major adverse cardiovascular events (MACE). This result is consistent with the findings of our review, which identified an increased risk of MACE in patients with PD. However, some reviews included in our analysis, such as Zhang et al. [42], presented heterogeneous results, highlighting the need for greater uniformity in the methodology of primary studies to improve comparability across studies.

An umbrella review conducted in 2023 [83], covering 31 SR on the association between PD and CVD, highlighted that to date, data on this association are heterogeneous and a definitive causal relationship cannot yet be established. Requiring further research, with properly designed long-term follow-up studies, to explore various pathophysiological aspects of this association.

Although most of the studies included in our review support the association between PD and CVD, the magnitude of this association varies considerably across studies [42, 84]. This variability may be influenced by factors such as differences in PD diagnostic criteria, heterogeneity in study populations, and the lack of control for confounding factors such as smoking and diabetes [84]. Therefore, it is essential that future studies adopt a standardized approach to the assessment of PD and consider the inclusion of sensitivity analyses to address these limitations [63].

Previous studies on the association between PD and CVD have been carried out mainly in cross-sectional, case–control, and cohort studies. Furthermore, research has also been conducted using intervention study designs to explore this association.

For example, López et al. [85] conducted a double-blind, parallel-arm randomized clinical trial to investigate whether periodontal therapy can decrease systemic inflammation in patients with metabolic syndrome and reduce cardiovascular risk. They concluded that reduction of periodontal inflammation, both through root scaling and systemic antibiotics and through plaque control and subgingival scaling, resulted in a significant decrease in C-reactive protein levels after nine months in this group of patients.

A recent SR [31] found that there is no reliable evidence on secondary prevention of CVD in patients with periodontitis and the evidence on primary prevention of CVD in this group of patients is very low and inconclusive regarding the effects of scaling and root planing with or without antibiotics compared to supragingival scaling.

In this study, a comprehensive literature search was carried out to summarize and analyze 41 available SR on the association between PD and CVD; however, these studies presented limitations related to the selected primary studies, because they differed in the types of study included and the definition criteria for PD (gingivitis or periodontitis), which complicated the performance of a meta-analysis.

Some studies in the analysis showed a high level of confidence, which could strengthen the evidence for the results and conclusions. However, the persistence of SR with lower confidence levels highlights the need for greater rigor in future research. The methodological quality of the SR identified deficiencies in critical domains such as the lack of an explicit statement on the review methods before execution, an incomplete literature search strategy, the absence of a list of excluded studies with justification, an unsatisfactory technique to assess the risk of bias in the included studies, and the lack of consideration of the risk of bias when interpreting or discussing the results.

It is important to be cautious when interpreting the results of the present study, as approximately 74% of the included primary studies are repeated in multiple reviews, which may distort the perception of the results. However, it would be beneficial to conduct new SR that take into consideration the methodological limitations identified by Moher [86] and in this review, given the high overlap between existing reviews.

### Evidence summary

In this umbrella review, we aimed to clarify the association between PD and CVD by collecting and analyzing relevant SR and meta-analyses on this topic. During this process, we identified the following key results:

The SR analyzed in this study support a positive and direct association between PD and CVD (CvD, ACVD, ACS, AF / AFL, AS, cardiac arrhythmias, CA, CAC, CAD, CaD, cardiac death, CHD, c–IMT / FMD, HT, stroke, LEAD, MACE, MI, PAD and PAOD). This result aligns with what was reported by Peruzzi et al. [83], who also found evidence of this association.

The association between PD and CVD was found to be more significant with increasing age. The most likely reason is that aging can have a degenerative effect on blood vessels, increasing the risk of developing CVDs such as MI and stroke [87, 88].

Furthermore, it was found that this association was more present in men. This could be explained by biological differences between men and women, such as hormone levels. Estrogens, hormones present at higher levels in women before menopause, may provide some cardiovascular protection [89].

And finally, the association was found to exist in most countries and continents. This is possibly due to globalization and the adoption of Western lifestyles in various regions, which has led to an increase in unhealthy eating habits, tobacco use, and lack of physical activity [87].

### Implications for clinical practice

Dental professionals bear the responsibility of raising awareness and providing education to patients regarding the link between PD and CVD. Promoting proper oral hygiene practices, such as regular brushing, flossing, and mouthwash use, can mitigate plaque accumulation and reduce CVD risks. In the realm of personalized healthcare, it is recommended to integrate periodontal assessments into routine risk evaluations, as well as to educate patients about CVD. It is crucial to implement preventive measures to modify risk factors and decrease the likelihood of both CVD and PD. Moreover, establishing a follow-up plan for patients with CVD, including regular dental check-ups and early PD detection, is vital. Collaborating with cardiologists, nutritionists, and other specialists enables a holistic approach to managing patients with CVD, facilitating coordinated medical and dental care.

### Implications for research

This review underscores the significance of enhancing the quality of SR presentation. The authors advocate for the utilization of quality assessment tools to inform the construction of future SR. They also stress the significance of conducting primary studies with robust methodological rigor to ensure the reliability of outcomes.

For forthcoming research in this domain, it is recommended to standardize diagnostic criteria for PD, undertake high-caliber prospective studies with substantial sample sizes and uniform measures, and conduct more comprehensive inquiries to elucidate the exact mechanisms and extent of the association between PD and CVD.

## Conclusions

The findings of systematic reviews with high overall confidence support the association between PD, tooth loss, and CVDs. However, it is crucial to interpret these results with caution due to the methodological limitations of the included studies, specifically those referenced in the systematic reviews. The potential public health relevance of this association justifies the implementation of oral health strategies that include both preventive and corrective interventions. Additionally, the need for more rigorous future research is emphasized to strengthen the evidence and guide the implementation of effective public health strategies.

## Supplementary Information

Supplementary Material 1. Overlapping of primary studies in systematic reviews.

## Data Availability

The dataset supporting the conclusions of this article is included within the article. However, additional information can be requested from the corresponding author upon reasonable inquiry.

## Abbreviations

PD: Periodontal Disease
PDs: Periodontal Diseases
CVD: Cardiovascular Disease
CVDs: Cardiovascular Diseases
SR: Systematic Review(s)
OR: Odds Ratio
RR: Risk Ratio
AMSTAR-2: A Measurement Tool to Assess Systematic Reviews
PRISMA-P: Preferred Reporting Items for Systematic Reviews and Meta-Analysis Protocols
PROSPERO: Prospective Registry of Systematic Reviews
GRADE: Grading of Recommendations Assessment, Development and Evaluation
HR: Hazard Ratio
MD: Mean Difference
CvD: Cerebrovascular Disease
ACVD: Atherosclerotic Cardiovascular Disease
ACS: Acute Coronary Syndrome
AF/AFL: Atrial Fibrillation / Atrial Flutter
AS: Arterial Stiffness
CA: Carotid Atherosclerosis
CAC: Carotid Artery Calcification
CAD: Coronary Artery Disease
CaD: Carotid Artery Disease
CHD: Coronary Heart Disease
c-IMT/FMD: Carotid Intima-Media Thickness / Flow-Mediated Dilation
HT: Hypertension
LEAD: Lower Extremity Arterial Disease
MACE: Major Adverse Cardiovascular Event
MI: Myocardial Infarction
PAD: Peripheral Artery Disease
PAOD: Peripheral Arterial Occlusive Disease
GTR: Guided tissue regeneration
