# Supplementary material for: Periodontal disease and cardiovascular disease: umbrella review
Source: BMC Oral Health. 2024 Oct 28;24:1308. doi: 10.1186/s12903-024-04907-1 (PMC11520879; doi:10.1186/s12903-024-04907-1)
Supplement: Supplementary file 1 — Supplementary Material 1. Overlapping of primary studies in systematic reviews. [file 12903_2024_4907_MOESM1_ESM.docx]

[**SUPPLEMENTARY MATERIAL**](https://static-content.springer.com/esm/art%3A10.1186%2Fs12903-023-03499-6/MediaObjects/12903_2023_3499_MOESM1_ESM.docx) **1.** Overlapping of primary studies in systematic reviews.

| **Primary Studies** | **Systematic Reviews That Included the Primary Studies** | **Times That Primary Studies Were Included** |
| --- | --- | --- |
| Howell et al. [1] | Alwithanani et al. [2], Guo et al. [3], Leng et al. [4], Meregildo-Rodríguez et al. [5], Qin et al. [6], Larvin et al. [7], Gao et al. [8], Cheng et al. [9], Xu et al. [10], Lafon et al. [11], Sfyroeras et al. [12], Humphrey et al. [13], Bahekar et al. [14], Khader et al. [15], Janket et al. [16], Madianos et al. [17] | 16 |
| Joshipura et al. [18] | Alwithanani et al. [2], Guo et al. [3], Leng et al. [4], Meregildo-Rodríguez et al. [5], Qin et al. [6], Beukers et al. [19], Larvin et al. [7], Gao et al. [8], Kumari et al.[20], Cheng et al. [9], Xu et al. [10], Bahekar et al. [14], Khader et al. [15], Janket et al. [16], Madianos et al. [17] | 15 |
| Morrison et al. [21] | Alwithanani et al. [2], Guo et al. [3], Leng et al. [4], Meregildo-Rodríguez et al. [5], Beukers et al. [19], Larvin et al. [7], Gao et al. [8], Kumari et al.[20], Lafon et al. [11], Blaizot et al. [22], Humphrey et al. [13], Khader et al. [15], Janket et al. [16], Madianos et al. [17] | 14 |
| De Stefano et al. [23] | Alwithanani et al. [2], Guo et al. [3], Leng et al. [4], Meregildo-Rodríguez et al. [5], Larvin et al. [7], Gao et al. [8], Blaizot et al. [22], Humphrey et al. [13], Bahekar et al. [14], Khader et al. [15], Janket et al. [16], Madianos et al. [17] | 12 |
| Beck et al. [24] | Alwithanani et al. [2], Guo et al. [3], Leng et al. [4], Meregildo-Rodríguez et al. [5], Larvin et al. [7], Kumari et al.[20], Leira et al. [25], Sfyroeras et al. [12], Humphrey et al. [13], Khader et al. [15], Janket et al. [16], Madianos et al. [17] | 12 |
| Hung et al. [26] | Alwithanani et al. [2], Guo et al. [3], Leng et al. [4], Beukers et al. [19], Larvin et al. [7], Gao et al. [8], Peng et al. [27], Cheng et al. [9], Polzer et al. [28], Blaizot et al. [22], Humphrey et al. [13] | 11 |
| Noguchi et al. [29] | Alwithanani et al. [2], Guo et al. [3], Leng et al. [4], Meregildo-Rodríguez et al. [5], Qin et al. [6], Beukers et al. [19], Larvin et al. [7], Gao et al. [8], Bodanese et al. [30], Cheng et al. [9] | 10 |
| Wu et al. [31] | Alwithanani et al. [2], Guo et al. [3], Beukers et al. [19], Larvin et al. [7], Fagundes et al. [32], Leira et al. [25], Lafon et al. [11], Sfyroeras et al. [12] Khader et al. [15] | 9 |
| Tuominen et al. [33] | Leng et al. [4], Beukers et al. [19], Aguilera et al. [34], Peng et al. [27], Cheng et al. [9], Dietrich et al. [35], Polzer et al. [28], Blaizot et al. [22], Humphrey et al. [13] | 9 |
| Abnet et al. [36] | Alwithanani et al. [2], Guo et al. [3], Beukers et al. [19], Larvin et al. [7], Cheng et al. [9], Lafon et al. [11], Polzer et al. [28], Sfyroeras et al. [12], Blaizot et al. [22] | 9 |
| Hujoel et al. [37] | Meregildo-Rodríguez et al. [5], Gao et al. [8], Aguilera et al. [34], Kumari et al.[20], Cheng et al. [9], Khader et al. [15], Janket et al. [16], Madianos et al. [17] | 8 |
| Tu et al. [38] | Alwithanani et al. [2], Guo et al. [3], Beukers et al. [19], Larvin et al. [7], Peng et al. [27], Cheng et al. [9], Lafon et al. [11], Polzer et al. [28] | 8 |
| Dietrich et al. [39] | Alwithanani et al. [2], Guo et al. [3], Leng et al. [4], Beukers et al. [19], Larvin et al. [7], Peng et al. [27], Cheng et al. [9], Dietrich et al. [35] | 8 |
| Jimenez et al. [40] | Alwithanani et al. [2], Guo et al. [3], Leng et al. [4], Larvin et al. [7], Leira et al. [25], Lafon et al. [11], Dietrich et al. [35], Sfyroeras et al. [12] | 8 |
| Yu et al. [41] | Alwithanani et al. [2], Guo et al. [3], Leng et al. [4], Meregildo-Rodríguez et al. [5], Qin et al. [6], Larvin et al. [7], Gao et al. [8], Xu et al. [10] | 8 |
| Arbes et al. [42] | Meregildo-Rodríguez et al. [5], Bodanese et al. [30], Kumari et al.[20], Blaizot et al. [22], Bahekar et al. [14], Khader et al. [15], Madianos et al. [17] | 7 |
| Joshipura et al. [43] | Alwithanani et al. [2], Guo et al. [3], Leng et al. [4], Beukers et al. [19], Larvin et al. [7], Lafon et al. [11], Sfyroeras et al. [12] | 7 |
| Holmlund et al. [44] | Beukers et al. [19], Gao et al. [8], Peng et al. [27], Fagundes et al. [32], Cheng et al. [9], Lafon et al. [11], Polzer et al. [28] | 7 |
| Rivas–Tumanyan et al. [45] | Alwithanani et al. [2], Leng et al. [4], Xu et al. [46], Tada et al. [47], Larvin et al. [7], Aguilera et al. [34], Martín-Cabezas et al. [48] | 7 |
| Hansen et al. [49] | Alwithanani et al. [2], Guo et al. [3], Leng et al. [4], Meregildo-Rodríguez et al. [5], Qin et al. [6], Larvin et al. [7], Bodanese et al. [30] | 7 |
| Joshy et al. [50] | Alwithanani et al. [2], Guo et al. [3], Leng et al. [4], Beukers et al. [19], Larvin et al. [7], Peng et al. [27], Cheng et al. [9] | 7 |
| Holmlund et al. [51] | Alwithanani et al. [2], Guo et al. [3], Meregildo-Rodríguez et al. [5], Qin et al. [6], Beukers et al. [19], Larvin et al. [7], Bodanese et al. [30] | 7 |
| Buhlin et al. [52] | Aguilera et al. [34], Xu et al. [10], Blaizot et al. [22], Bahekar et al. [14], Khader et al. [15], Madianos et al. [17] | 6 |
| Hung et al. [53] | Alwithanani et al. [2], Leng et al. [4], Beukers et al. [19], Larvin et al. [7], Kaschwich et al. [54], Wang et al. [55] | 6 |
| Holmlund et al. [56] | Meregildo-Rodríguez et al. [5], Bodanese et al. [30], Aguilera et al. [34], Xu et al. [10], Martín-Cabezas et al. [48], Blaizot et al. [22] | 6 |
| Heitmann et al. [57] | Alwithanani et al. [2], Guo et al. [3], Leng et al. [4], Beukers et al. [19], Larvin et al. [7], Lafon et al. [11] | 6 |
| Choe et al. [58] | Alwithanani et al. [2], Guo et al. [3], Leng et al. [4], Xu et al. [46], Beukers et al. [19], Larvin et al. [7] | 6 |
| Liljestrand et al. [59] | Guo et al. [3], Meregildo-Rodríguez et al. [5], Qin et al. [6], Beukers et al. [19], Peng et al. [27], Cheng et al. [9] | 6 |
| Chen et al. [60] | Alwithanani et al. [2], Leelaviwat et al. [61], Zhang et al. [62], Leng et al. [4], Leelapatana et al. [63], Larvin et al. [7] | 6 |
| La Monte et al. [64] | Alwithanani et al. [2], Guo et al. [3], Leng et al. [4], Beukers et al. [19], Larvin et al. [7], Kumari et al.[20] | 6 |
| Cueto et al. [65] | Meregildo-Rodríguez et al. [5], Xu et al. [10], Shi et al. [66], Dietrich et al. [35], Blaizot et al. [22] | 5 |
| Andriankaja et al. [67] | Leng et al. [4], Meregildo-Rodríguez et al. [5], Xu et al. [10], Shi et al. [66], Dietrich et al. [35] | 5 |
| Mucci et al. [68] | Alwithanani et al. [2], Guo et al. [3], Beukers et al. [19], Larvin et al. [7], Gao et al. [8] | 5 |
| Dorn et al. [69] | Guo et al. [3], Meregildo-Rodríguez et al. [5], Qin et al. [6], Xu et al. [10], Dietrich et al. [35] | 5 |
| Lee et al. [70] | Alwithanani et al. [2], Guo et al. [3], Meregildo-Rodríguez et al. [5], Qin et al. [6], Larvin et al. [7] | 5 |
| Lee et al. [71] | Alwithanani et al. [2], Guo et al. [3], Meregildo-Rodríguez et al. [5], Larvin et al. [7], Aguilera et al. [34] | 5 |
| Batty et al. [72] | Alwithanani et al. [2], Guo et al. [3], Leng et al. [4], Beukers et al. [19], Larvin et al. [7] | 5 |
| Mendez et al. [73] | Kaschwich et al. [54], Wang et al. [55], Yang et al. [74], Dietrich et al. [35] | 4 |
| Loesche et al. [75] | Blaizot et al. [22], Bahekar et al. [14], Khader et al. [15], Madianos et al. [17] | 4 |
| Wu et al. [76] | Meregildo-Rodríguez et al. [5], Gao et al. [8], Bahekar et al. [14], Janket et al. [16] | 4 |
| Hujoel et al. [77] | Alwithanani et al. [2], Guo et al. [3], Larvin et al. [7], Madianos et al. [17] | 4 |
| Rutger Persson et al. [78] | Meregildo-Rodríguez et al. [5], Xu et al. [10], Shi et al. [66], Blaizot et al. [22] | 4 |
| Cabrera et al. [79] | Beukers et al. [19], Peng et al. [27], Polzer et al. [28], Blaizot et al. [22] | 4 |
| Andriankaja et al. [80] | Meregildo-Rodríguez et al. [5], Xu et al. [10], Shi et al. [66], Blaizot et al. [22] | 4 |
| Lee et al. [81] | Alwithanani et al. [2], Guo et al. [3], Leng et al. [4], Larvin et al. [7] | 4 |
| Lee et al. [82] | Meregildo-Rodríguez et al. [5], Bodanese et al. [30], Aguilera et al. [34], Martín-Cabezas et al. [48] | 4 |
| Sen et al. [83] | Alwithanani et al. [2], Guo et al. [3], Larvin et al. [7], Fagundes et al. [32] | 4 |
| Lee et al. [84] | Guo et al. [3], Meregildo-Rodríguez et al. [5], Qin et al. [6], Beukers et al. [19] | 4 |
| Mattila et al. [85] | Bahekar et al. [14], Janket et al. [16], Madianos et al. [17] | 3 |
| Emingil et al. [86] | Kumari et al.[20], Shi et al. [66], Madianos et al. [17] | 3 |
| Beck et al. [87] | Wang et al. [55], Zeng et al. [88], Orlandi et al. [89] | 3 |
| Katz et al. [90] | Kumari et al.[20], Blaizot et al. [22], Madianos et al. [17] | 3 |
| Bloemenkamp et al. [91] | Kaschwich et al. [54], Wang et al. [55], Yang et al. [74] | 3 |
| Persson et al. [92] | Wang et al. [55], Zeng et al. [88], Madianos et al. [17] | 3 |
| Ravon et al. [93] | Wang et al. [94], Wang et al. [55], Zeng et al. [88] | 3 |
| Ajwani et al. [95] | Beukers et al. [19], Dietrich et al. [35], Humphrey et al. [13] | 3 |
| Dörfer et al. [96] | Fagundes et al. [32], Leira et al. [25], Sfyroeras et al. [12] | 3 |
| Grau et al. [97] | Leira et al. [25], Dietrich et al. [35], Sfyroeras et al. [12] | 3 |
| Geerts et al. [98] | Meregildo-Rodríguez et al. [5], Blaizot et al. [22], Bahekar et al. [14] | 3 |
| Elter et al. [99] | Cheng et al. [9], Blaizot et al. [22], Bahekar et al. [14] | 3 |
| Söder et al. [100] | Wang et al. [55], Zeng et al. [88], Orlandi et al. [89] | 3 |
| Buhlin et al. [101] | Meregildo-Rodríguez et al. [5], Blaizot et al. [22], Bahekar et al. [14] | 3 |
| Briggs et al. [102] | Meregildo-Rodríguez et al. [5], Blaizot et al. [22], Bahekar et al. [14] | 3 |
| Padilha et al. [103] | Beukers et al. [19], Peng et al. [27], Polzer et al. [28] | 3 |
| Österberg et al. [104] | Beukers et al. [19], Peng et al. [27], Polzer et al. [28] | 3 |
| Chen et al. [105] | Kaschwich et al. [54], Wang et al. [55], Yang et al. [74] | 3 |
| Sim et al. [106] | Leira et al. [25], Dietrich et al. [35], Sfyroeras et al. [12] | 3 |
| Pradeep et al. [107] | Fagundes et al. [32], Leira et al. [25], Sfyroeras et al. [12] | 3 |
| Holmlund et al. [108] | Meregildo-Rodríguez et al. [5], Xu et al. [10], Shi et al. [66] | 3 |
| Vieira et al. [109] | Aguilera et al. [34], Schmitt et al. [110], Orlandi et al. [89] | 3 |
| López – Jornet et al. [111] | Wang et al. [55], Zeng et al. [88], Orlandi et al. [89] | 3 |
| Schwahn et al. [112] | Beukers et al. [19], Peng et al. [27], Cheng et al. [9] | 3 |
| Soto–Barreras et al. [113] | Kaschwich et al. [54], Wang et al. [55], Yang et al. [74] | 3 |
| Khosravi et al. [114] | Meregildo-Rodríguez et al. [5], Xu et al. [10], Shi et al. [66] | 3 |
| Wozakowska et al. [115] | Wang et al. [55], Shi et al. [66], Zeng et al. [88] | 3 |
| Hayashida et al. [116] | Wang et al. [55], Zeng et al. [88], Orlandi et al. [89] | 3 |
| Pinho et al. [117] | Wang et al. [55], Zeng et al. [88], Orlandi et al. [89] | 3 |
| Iwashima et al. [118] | Xu et al. [46], Aguilera et al. [34], Martín-Cabezas et al. [48] | 3 |
| Jung et al. [119] | Wang et al. [55], Cheng et al. [9], Zeng et al. [88] | 3 |
| Willershausen et al. [120] | Meregildo-Rodríguez et al. [5], Xu et al. [10], Shi et al. [66] | 3 |
| Kodovazenitis et al. [121] | Meregildo-Rodríguez et al. [5], Xu et al. [10], Shi et al. [66] | 3 |
| Chou et al. [122] | Alwithanani et al. [2], Guo et al. [3], Larvin et al. [7] | 3 |
| Darnaud et al. [123] | Xu et al. [46], Tada et al. [47], Martín-Cabezas et al. [48] | 3 |
| Ahn et al. [124] | Leng et al. [4], Aguilera et al. [34], Martín-Cabezas et al. [48] | 3 |
| Vedin et al. [125] | Beukers et al. [19], Peng et al. [27], Cheng et al. [9] | 3 |
| Ahn et al. [126] | Kaschwich et al. [54], Wang et al. [55], Yang et al. [74] | 3 |
| Rydén et al. [127] | Meregildo-Rodríguez et al. [5], Xu et al. [10], Shi et al. [66] | 3 |
| Çalapkorur et al. [128] | Kaschwich et al. [54], Wang et al. [55], Yang et al. [74] | 3 |
| Lin et al. [129] | Alwithanani et al. [2], Guo et al. [3], Larvin et al. [7] | 3 |
| Chang et al. [130] | Zhang et al. [62], Leelapatana et al. [63], Beukers et al. [19] | 3 |
| Sen et al. [131] | Leelaviwat et al. [61], Zhang et al. [62], Leelapatana et al. [63] | 3 |
| Shimazaki et al. [132] | Beukers et al. [19], Polzer et al. [28] | 2 |
| Jansson et al. [133] | Kumari et al.[20] Madianos et al. [17] | 2 |
| Bazile et al. [134] | Meregildo-Rodríguez et al. [5], Xu et al. [10] | 2 |
| Malthaner et al. [135] | Blaizot et al. [22], Bahekar et al. [14] | 2 |
| López et al. [136] | Dietrich et al. [35], Madianos et al. [17] | 2 |
| Buhlin et al. [137] | Aguilera et al. [34], Martín-Cabezas et al. [48] | 2 |
| Hämäläinen et al. [138] | Beukers et al. [19], Polzer et al. [28] | 2 |
| Elter et al. [139] | Guo et al. [3], Sfyroeras et al. [12] | 2 |
| Buhlin et al. [140] | Blaizot et al. [22], Bahekar et al. [14] | 2 |
| Desvarieux et al. [141] | Zeng et al. [88], Orlandi et al. [89] | 2 |
| Ragnarsson et al. [142] | Beukers et al. [19], Polzer et al. [28] | 2 |
| Renvert et al. [143] | Shi et al. [66], Blaizot et al. [22] | 2 |
| Montebugnoli et al. [144] | Meregildo-Rodríguez et al. [5], Blaizot et al. [22] | 2 |
| Engebretson et al. [145] | Wang et al. [55], Zeng et al. [88] | 2 |
| Leivadaros et al. [146] | Wang et al. [55], Orlandi et al. [89] | 2 |
| Beck et al. [147] | Leng et al. [4], Orlandi et al. [89] | 2 |
| Desvarieux et al. [148] | Wang et al. [55], Orlandi et al. [89] | 2 |
| Seinost et al. [149] | Aguilera et al. [34], Orlandi et al. [89] | 2 |
| Hämäläinen et al. [150] | Peng et al. [27], Polzer et al. [28] | 2 |
| Coelho et al. [151] | Meregildo-Rodríguez et al. [5], Blaizot et al. [22] | 2 |
| Völzke et al. [152] | Xu et al. [46], Tada et al. [47] | 2 |
| Schillinger et al. [153] | Wang et al. [55], Zeng et al. [88] | 2 |
| Morita et al. [154] | Beukers et al. [19], Polzer et al. [28] | 2 |
| Geismar et al. [155] | Meregildo-Rodríguez et al. [5], Blaizot et al. [22] | 2 |
| Spahr et al. [156] | Meregildo-Rodríguez et al. [5], Blaizot et al. [22] | 2 |
| Engström et al. [157] | Aguilera et al. [34], Martín-Cabezas et al. [48] | 2 |
| Tonetti et al. [158] | Aguilera et al. [34], Orlandi et al. [89] | 2 |
| Österberg et al. [159] | Beukers et al. [19], Polzer et al. [28] | 2 |
| Nonnemacher et al. [160] | Meregildo-Rodríguez et al. [5], Blaizot et al. [22] | 2 |
| Rech et al. [161] | Meregildo-Rodríguez et al. [5], Blaizot et al. [22] | 2 |
| Senba et al. [162] | Leng et al. [4], Xu et al. [10] | 2 |
| Higashi et al. [163] | Aguilera et al. [34], Orlandi et al. [89] | 2 |
| Holm–Pedersen et al. [164] | Beukers et al. [19], Polzer et al. [28] | 2 |
| Fukai et al. [165] | Beukers et al. [19], Polzer et al. [28] | 2 |
| Franek et al. [166] | Aguilera et al. [34], Schmitt et al. [110] | 2 |
| Brown et al. [167] | Beukers et al. [19], Polzer et al. [28] | 2 |
| Higashi et al. [168] | Aguilera et al. [34], Orlandi et al. [89] | 2 |
| Carallo et al.[169] | Wang et al. [55], Zeng et al. [88] | 2 |
| Tsakos et al. [170] | Aguilera et al. [34], Martín-Cabezas et al. [48] | 2 |
| Nesse et al. [171] | Aguilera et al. [34], Martín-Cabezas et al. [48] | 2 |
| Desvarieux et al. [172] | Aguilera et al. [34], Martín-Cabezas et al. [48] | 2 |
| Islas–Granillo et al. [173] | Xu et al. [46], Tada et al. [47] | 2 |
| Paganini–Hill et al. [174] | Beukers et al. [19], Peng et al. [27] | 2 |
| Yamori et al. [175] | Aguilera et al. [34], Martín-Cabezas et al. [48] | 2 |
| Vidal et al. [176] | Aguilera et al. [34], Martín-Cabezas et al. [48] | 2 |
| Watt et al. [177] | Beukers et al. [19], Cheng et al. [9] | 2 |
| Franek et al. [178] | Schmitt et al. [110], Orlandi et al. [89] | 2 |
| Chen et al. [179] | Zhang et al. [62], Leelapatana et al. [63] | 2 |
| Hayasaka et al. [180] | Beukers et al. [19], Peng et al. [27] | 2 |
| Parkar et al. [181] | Meregildo-Rodríguez et al. [5], Xu et al. [10] | 2 |
| Li et al. [182] | Meregildo-Rodríguez et al. [5], Xu et al. [10] | 2 |
| Ramesh et al. [183] | Wang et al. [55], Zeng et al. [88] | 2 |
| Rivas–Tumanyan et al. [184] | Aguilera et al. [34], Martín-Cabezas et al. [48] | 2 |
| Vidal et al. [185] | Aguilera et al. [34], Schmitt et al. [110] | 2 |
| Janket et al. [186] | Beukers et al. [19], Peng et al. [27] | 2 |
| Ando et al. [187] | Beukers et al. [19], Peng et al. [27] | 2 |
| Yu et al. [188] | Wang et al. [55], Zeng et al. [88] | 2 |
| Machida et al. [189] | Aguilera et al. [34], Martín-Cabezas et al. [48] | 2 |
| Ollikainen et al. [190] | Aguilera et al. [34], Martín-Cabezas et al. [48] | 2 |
| Zhu et al. [191] | Xu et al. [46], Tada et al. [47] | 2 |
| Hu et al. [192] | Beukers et al. [19], Peng et al. [27] | 2 |
| Choi et al. [193] | Aguilera et al. [34], Martín-Cabezas et al. [48] | 2 |
| Laguzzi et al. [194] | Xu et al. [46], Tada et al. [47] | 2 |
| Kim et al. [195] | Xu et al. [46], Tada et al. [47] | 2 |
| Singh et al. [196] | Xu et al. [46], Tada et al. [47] | 2 |
| Moghadam et al. [197] | Tada et al. [47], Aguilera et al. [34] | 2 |
| Górski et al. [198] | Meregildo-Rodríguez et al. [5], Aguilera et al. [34] | 2 |
| Lysek et al. [199] | Aguilera et al. [34], Martín-Cabezas et al. [48] | 2 |
| Kawabata et al. [200] | Aguilera et al. [34], Martín-Cabezas et al. [48] | 2 |
| Chrysanthakooulos et al. [201] | Aguilera et al. [34], Martín-Cabezas et al. [48] | 2 |
| Muñoz–Torres et al. [202] | Beukers et al. [19], Kaschwich et al. [54] | 2 |
| Aoyama et al. [203] | Wang et al. [55], Yang et al. [74] | 2 |
| Im et al. [204] | Zhang et al. [62], Leelapatana et al. [63] | 2 |
| Shin et al. [205] | Xu et al. [46], Tada et al. [47] | 2 |
| Gordon et al. [206] | Tada et al. [47], Aguilera et al. [34] | 2 |
| Da et al. [207] | Xu et al. [46], Tada et al. [47] | 2 |
| Gordon et al. [208] | Xu et al. [46], Tada et al. [47] | 2 |
| Qi et al. [209] | Guo et al. [3], Beukers et al. [19] | 2 |
| Mendes et al. [210] | Xu et al. [46], Tada et al. [47] | 2 |
| Woo et al. [211] | Xu et al. [46], Tada et al. [47] | 2 |
| Hsu et al. [212] | Leelaviwat et al. [61], Zhang et al. [62] | 2 |

References

1. Howell, T.H.; Ridker, P.M.; Ajani, U.A.; Hennekens, C.H.; Christen, W.G. Periodontal Disease and Risk of Subsequent Cardiovascular Disease in U.S. Male Physicians. *J. Am. Coll. Cardiol.* **2001**, *37*, 445–450, doi:10.1016/s0735-1097(00)01130-x.

2. Alwithanani, N. Periodontal Diseases and Heart Diseases: A Systemic Review. *J. Pharm. Bioallied Sci.* **2023**, *15*, 72–78, doi:10.4103/jpbs.jpbs_517_22.

3. Guo, X.; Li, X.; Liao, C.; Feng, X.; He, T. Periodontal Disease and Subsequent Risk of Cardiovascular Outcome and All-Cause Mortality: A Meta-Analysis of Prospective Studies. *Plos One* **2023**, *18*, e0290545.

4. Leng, Y.; Hu, Q.; Ling, Q.; Yao, X.; Liu, M.; Chen, J.; Yan, Z.; Dai, Q. Periodontal Disease Is Associated with the Risk of Cardiovascular Disease Independent of Sex: A Meta-Analysis. *Front. Cardiovasc. Med.* **2023**, *10*, 1114927, doi:10.3389/fcvm.2023.1114927.

5. Meregildo-Rodriguez, E.D.; Robles-Arce, L.G.; Chunga-Chévez, E.V.; Asmat-Rubio, M.G.; Zavaleta-Alaya, P.; Vásquez-Tirado, G.A. Periodontal Disease as a Non-Traditional Risk Factor for Acute Coronary Syndrome: A Systematic Review and Meta-Analysis. *Infez. Med.* **2022**, *30*, 501–515, doi:10.53854/liim-3004-4.

6. Qin, X.; Zhao, Y.; Guo, Y. Periodontal Disease and Myocardial Infarction Risk: A Meta-Analysis of Cohort Studies. *Am. J. Emerg. Med.* **2021**, *48*, 103–109, doi:10.1016/j.ajem.2021.03.071.

7. Larvin, H.; Kang, J.; Aggarwal, V.R.; Pavitt, S.; Wu, J. Risk of Incident Cardiovascular Disease in People with Periodontal Disease: A Systematic Review and Meta-Analysis. *Clin. Exp. Dent. Res.* **2021**, *7*, 109–122, doi:10.1002/cre2.336.

8. Gao, S.; Tian, J.; Li, Y.; Liu, T.; Li, R.; Yang, L.; Xing, Z. Periodontitis and Number of Teeth in the Risk of Coronary Heart Disease: An Updated Meta-Analysis. *Med. Sci. Mmonitor* **2021**, *27*, doi:10.12659/MSM.930112.

9. Cheng, F.; Zhang, M.; Wang, Q.; Xu, H.; Dong, X.; Gao, Z.; Chen, J.; Wei, Y.; Qin, F. Tooth Loss and Risk of Cardiovascular Disease and Stroke: A Dose-Response Meta Analysis of Prospective Cohort Studies. *PLoS ONE* **2018**, *13*, doi:10.1371/journal.pone.0194563.

10. Xu, S.; Song, M.; Xiong, Y.; Liu, X.; He, Y.; Qin, Z. The Association between Periodontal Disease and the Risk of Myocardial Infarction: A Pooled Analysis of Observational Studies. *BMC Cardiovasc. Disord.* **2017**, *17*, 50, doi:10.1186/s12872-017-0480-y.

11. Lafon, A.; Pereira, B.; Dufour, T.; Rigouby, V.; Giroud, M.; Béjot, Y.; Tubert-Jeannin, S. Periodontal Disease and Stroke: A Meta-Analysis of Cohort Studies. *Eur. J. Neurol.* **2014**, *21*, 1155–1161, doi:10.1111/ene.12415.

12. Sfyroeras, G.S.; Roussas, N.; Saleptsis, V.G.; Argyriou, C.; Giannoukas, A.D. Association between Periodontal Disease and Stroke. *J. Vasc. Surg.* **2012**, *55*, 1178–1184, doi:10.1016/j.jvs.2011.10.008.

13. Humphrey, L.L.; Fu, R.; Buckley, D.I.; Freeman, M.; Helfand, M. Periodontal Disease and Coronary Heart Disease Incidence: A Systematic Review and Meta-Analysis. *J. Gen. Intern. Med.* **2008**, *23*, 2079–2086, doi:10.1007/s11606-008-0787-6.

14. Bahekar, A.A.; Singh, S.; Saha, S.; Molnar, J.; Arora, R. The Prevalence and Incidence of Coronary Heart Disease Is Significantly Increased in Periodontitis: A Meta-Analysis. *Am. Heart J.* **2007**, *154*, 830–837, doi:10.1016/j.ahj.2007.06.037.

15. Khader, Y.S.; Albashaireh, Z.S.M.; Alomari, M.A. Periodontal Diseases and the Risk of Coronary Heart and Cerebrovascular Diseases: A Meta-Analysis. *J. Periodontol.* **2004**, *75*, 1046–1053, doi:10.1902/jop.2004.75.8.1046.

16. Janket, S.; Baird, A.; Chuang, S.; Jones, J. Meta-Analysis of Periodontal Disease and Risk of Coronary Heart Disease and Stroke. *Oral Surg. Oral Med. Oral Pathol. Oral Radiol. Endod.* **2003**, *95*, 559–569, doi:10.1067/moe.2003.107.

17. Madianos, P.N.; Bobetsis, G.A.; Kinane, D.F. Is Periodontitis Associated with an Increased Risk of Coronary Heart Disease and Preterm and/or Low Birth Weight Births? *J. Clin. Periodontol.* **2002**, *29 Suppl 3*, 22–36; discussion 37-38, doi:10.1034/j.1600-051x.29.s3.2.x.

18. Joshipura, K.J.; Rimm, E.B.; Douglass, C.W.; Trichopoulos, D.; Ascherio, A.; Willett, W.C. Poor Oral Health and Coronary Heart Disease. *J. Dent. Res.* **1996**, *75*, 1631–1636, doi:10.1177/00220345960750090301.

19. Beukers, N.G.F.M.; Su, N.; Loos, B.G.; van der Heijden, G.J.M.G. Lower Number of Teeth Is Related to Higher Risks for ACVD and Death-Systematic Review and Meta-Analyses of Survival Data. *Front. Cardiovasc. Med.* **2021**, *8*, 621626, doi:10.3389/fcvm.2021.621626.

20. Kumari, M.; Patthi, B.; Jankiram, C.; Singla, A.; Malhi, R.; Rajeev, A. Risk of Having Myocardial Infarction in Periodontal Disease Patients: A Systematic Review. *J. Indian Assoc. Public Health Dent.* **2019**, *17*, 174–180, doi:10.4103/jiaphd.jiaphd_15_19.

21. Morrison, H.I.; Ellison, L.F.; Taylor, G.W. Periodontal Disease and Risk of Fatal Coronary Heart and Cerebrovascular Diseases. *J. Cardiovasc. Risk* **1999**, *6*, 7–11, doi:10.1177/204748739900600102.

22. Blaizot, A.; Vergnes, J.; Nuwwareh, S.; Amar, J.; Sixou, M. Periodontal Diseases and Cardiovascular Events: Meta-Analysis of Observational Studies. *Int. Dent. J.* **2009**, *59*, 197–209, doi:10.1922/IDJ_2114Sixou13.

23. DeStefano, F.; Anda, R.F.; Kahn, H.S.; Williamson, D.F.; Russell, C.M. Dental Disease and Risk of Coronary Heart Disease and Mortality. *BMJ* **1993**, *306*, 688–691, doi:10.1136/bmj.306.6879.688.

24. Beck, J.; Garcia, R.; Heiss, G.; Vokonas, P.S.; Offenbacher, S. Periodontal Disease and Cardiovascular Disease. *J. Periodontol.* **1996**, *67*, 1123–1137, doi:10.1902/jop.1996.67.10s.1123.

25. Leira, Y.; Seoane, J.; Blanco, M.; Rodríguez-Yáñez, M.; Takkouche, B.; Blanco, J.; Castillo, J. Association between Periodontitis and Ischemic Stroke: A Systematic Review and Meta-Analysis. *Eur. J. Epidemiol.* **2017**, *32*, 43–53, doi:10.1007/s10654-016-0170-6.

26. Hung, H.-C.; Joshipura, K.J.; Colditz, G.; Manson, J.E.; Rimm, E.B.; Speizer, F.E.; Willett, W.C. The Association between Tooth Loss and Coronary Heart Disease in Men and Women. *J. Public Health Dent.* **2004**, *64*, 209–215, doi:10.1111/j.1752-7325.2004.tb02755.x.

27. Peng, J.; Song, J.; Han, J.; Chen, Z.; Yin, X.; Zhu, J.; Song, J. The Relationship between Tooth Loss and Mortality from All Causes, Cardiovascular Diseases, and Coronary Heart Disease in the General Population: Systematic Review and Dose-Response Meta-Analysis of Prospective Cohort Studies. *Biosci. Rep.* **2019**, *39*, BSR20181773, doi:10.1042/BSR20181773.

28. Polzer, I.; Schwahn, C.; Völzke, H.; Mundt, T.; Biffar, R. The Association of Tooth Loss with All-Cause and Circulatory Mortality. Is There a Benefit of Replaced Teeth? A Systematic Review and Meta-Analysis. *Clin. Oral Investig.* **2012**, *16*, 333–351, doi:10.1007/s00784-011-0625-9.

29. Noguchi, S.; Toyokawa, S.; Miyoshi, Y.; Suyama, Y.; Inoue, K.; Kobayashi, Y. Five-Year Follow-up Study of the Association between Periodontal Disease and Myocardial Infarction among Japanese Male Workers: MY Health Up Study. *J. Public Health Oxf. Engl.* **2015**, *37*, 605–611, doi:10.1093/pubmed/fdu076.

30. Bodanese, L.C.; Louzeiro, G.C.; Magnus, G.A.; Baptista, Â.H.; Salum, F.G.; Mattiello, R. Association between Periodontitis and Myocardial Infarction: Systematic Review and Meta-Analysis. *Int. J. Cardiovasc. Sci.* **2021**, *34*, 121–127, doi:10.36660/ijcs.20200055.

31. Wu, T.; Trevisan, M.; Genco, R.J.; Dorn, J.P.; Falkner, K.L.; Sempos, C.T. Periodontal Disease and Risk of Cerebrovascular Disease: The First National Health and Nutrition Examination Survey and Its Follow-up Study. *Arch. Intern. Med.* **2000**, *160*, 2749–2755, doi:10.1001/archinte.160.18.2749.

32. Fagundes, N.; Almeida, A.; Vilhena, K.; Magno, M.; Maia, L.; Lima, R. Periodontitis As A Risk Factor For Stroke: A Systematic Review And Meta-Analysis. *Vasc. Health Risk Manag.* **2019**, *15*, 519–532, doi:10.2147/VHRM.S204097.

33. Tuominen, R.; Reunanen, A.; Paunio, M.; Paunio, I.; Aromaa, A. Oral Health Indicators Poorly Predict Coronary Heart Disease Deaths. *J. Dent. Res.* **2003**, *82*, 713–718, doi:10.1177/154405910308200911.

34. Aguilera, E.; Suvan, J.; Buti, J.; Czesnikiewicz-Guzik, M.; Ribeiro, A.; Orlandi, M.; Guzik, T.; Hingorani, A.; Nart, J.; D’Aiuto, F. Periodontitis Is Associated with Hypertension: A Systematic Review and Meta-Analysis. *Cardiovasc. Res.* **2020**, *116*, 28–39, doi:10.1093/cvr/cvz201.

35. Dietrich, T.; Sharma, P.; Walter, C.; Weston, P.; Beck, J. The Epidemiological Evidence behind the Association between Periodontitis and Incident Atherosclerotic Cardiovascular Disease. *J. Clin. Periodontol.* **2013**, *40*, S70–S84, doi:10.1111/jcpe.12062.

36. Abnet, C.C.; Qiao, Y.-L.; Dawsey, S.M.; Dong, Z.-W.; Taylor, P.R.; Mark, S.D. Tooth Loss Is Associated with Increased Risk of Total Death and Death from Upper Gastrointestinal Cancer, Heart Disease, and Stroke in a Chinese Population-Based Cohort. *Int. J. Epidemiol.* **2005**, *34*, 467–474, doi:10.1093/ije/dyh375.

37. Hujoel, P.P.; Drangsholt, M.; Spiekerman, C.; DeRouen, T.A. Periodontal Disease and Coronary Heart Disease Risk. *JAMA* **2000**, *284*, 1406–1410, doi:10.1001/jama.284.11.1406.

38. Tu, Y.-K.; Galobardes, B.; Smith, G.D.; McCarron, P.; Jeffreys, M.; Gilthorpe, M.S. Associations between Tooth Loss and Mortality Patterns in the Glasgow Alumni Cohort. *Heart Br. Card. Soc.* **2007**, *93*, 1098–1103, doi:10.1136/hrt.2006.097410.

39. Dietrich, T.; Jimenez, M.; Krall Kaye, E.A.; Vokonas, P.S.; Garcia, R.I. Age-Dependent Associations between Chronic Periodontitis/Edentulism and Risk of Coronary Heart Disease. *Circulation* **2008**, *117*, 1668–1674, doi:10.1161/CIRCULATIONAHA.107.711507.

40. Jimenez, M.; Krall, E.A.; Garcia, R.I.; Vokonas, P.S.; Dietrich, T. Periodontitis and Incidence of Cerebrovascular Disease in Men. *Ann. Neurol.* **2009**, *66*, 505–512, doi:10.1002/ana.21742.

41. Yu, Y.-H.; Chasman, D.I.; Buring, J.E.; Rose, L.; Ridker, P.M. Cardiovascular Risks Associated with Incident and Prevalent Periodontal Disease. *J. Clin. Periodontol.* **2015**, *42*, 21–28, doi:10.1111/jcpe.12335.

42. Arbes, S.J.; Slade, G.D.; Beck, J.D. Association between Extent of Periodontal Attachment Loss and Self-Reported History of Heart Attack: An Analysis of NHANES III Data. *J. Dent. Res.* **1999**, *78*, 1777–1782, doi:10.1177/00220345990780120301.

43. Joshipura, K.J.; Hung, H.-C.; Rimm, E.B.; Willett, W.C.; Ascherio, A. Periodontal Disease, Tooth Loss, and Incidence of Ischemic Stroke. *Stroke* **2003**, *34*, 47–52, doi:10.1161/01.str.0000052974.79428.0c.

44. Holmlund, A.; Holm, G.; Lind, L. Number of Teeth as a Predictor of Cardiovascular Mortality in a Cohort of 7,674 Subjects Followed for 12 Years. *J. Periodontol.* **2010**, *81*, 870–876, doi:10.1902/jop.2010.090680.

45. Rivas-Tumanyan, S.; Spiegelman, D.; Curhan, G.C.; Forman, J.P.; Joshipura, K.J. Periodontal Disease and Incidence of Hypertension in the Health Professionals Follow-up Study. *Am. J. Hypertens.* **2012**, *25*, 770–776, doi:10.1038/ajh.2012.32.

46. Xu, K.; Yu, W.; Li, Y.; Li, Y.; Wan, Q.; Chen, L.; Dong, Y.; Tay, F.R.; Niu, L. Association between Tooth Loss and Hypertension: A Systematic Review and Meta-Analysis. *J. Dent.* **2022**, *123*, 104178, doi:10.1016/j.jdent.2022.104178.

47. Tada, A.; Tano, R.; Miura, H. The Relationship between Tooth Loss and Hypertension: A Systematic Review and Meta-Analysis. *Sci. Rep.* **2022**, *12*, 13311, doi:10.1038/s41598-022-17363-0.

48. Martin-Cabezas, R.; Seelam, N.; Petit, C.; Agossa, K.; Gaertner, S.; Tenenbaum, H.; Davideau, J.-L.; Huck, O. Association between Periodontitis and Arterial Hypertension: A Systematic Review and Meta-Analysis. *Am. Heart J.* **2016**, *180*, 98–112, doi:10.1016/j.ahj.2016.07.018.

49. Hansen, G.M.; Egeberg, A.; Holmstrup, P.; Hansen, P.R. Relation of Periodontitis to Risk of Cardiovascular and All-Cause Mortality (from a Danish Nationwide Cohort Study). *Am. J. Cardiol.* **2016**, *118*, 489–493, doi:10.1016/j.amjcard.2016.05.036.

50. Joshy, G.; Arora, M.; Korda, R.J.; Chalmers, J.; Banks, E. Is Poor Oral Health a Risk Marker for Incident Cardiovascular Disease Hospitalisation and All-Cause Mortality? Findings from 172 630 Participants from the Prospective 45 and Up Study. *BMJ Open* **2016**, *6*, e012386, doi:10.1136/bmjopen-2016-012386.

51. Holmlund, A.; Lampa, E.; Lind, L. Oral Health and Cardiovascular Disease Risk in a Cohort of Periodontitis Patients. *Atherosclerosis* **2017**, *262*, 101–106, doi:10.1016/j.atherosclerosis.2017.05.009.

52. Buhlin, K.; Gustafsson, A.; Håkansson, J.; Klinge, B. Oral Health and Cardiovascular Disease in Sweden. *J. Clin. Periodontol.* **2002**, *29*, 254–259, doi:10.1034/j.1600-051x.2002.290312.x.

53. Hung, H.-C.; Willett, W.; Merchant, A.; Rosner, B.A.; Ascherio, A.; Joshipura, K.J. Oral Health and Peripheral Arterial Disease. *Circulation* **2003**, *107*, 1152–1157, doi:10.1161/01.cir.0000051456.68470.c8.

54. Kaschwich, M.; Behrendt, C.-A.; Heydecke, G.; Bayer, A.; Debus, E.S.; Seedorf, U.; Aarabi, G. The Association of Periodontitis and Peripheral Arterial Occlusive Disease—A Systematic Review. *Int. J. Mol. Sci.* **2019**, *20*, doi:10.3390/ijms20122936.

55. Wang, J.; Geng, X.; Sun, J.; Zhang, S.; Yu, W.; Zhang, X.; Liu, H. The Risk of Periodontitis for Peripheral Vascular Disease: A Systematic Review. *Rev. Cardiovasc. Med.* **2019**, *20*, 81–89, doi:10.31083/j.rcm.2019.02.52.

56. Holmlund, A.; Holm, G.; Lind, L. Severity of Periodontal Disease and Number of Remaining Teeth Are Related to the Prevalence of Myocardial Infarction and Hypertension in a Study Based on 4,254 Subjects. *J. Periodontol.* **2006**, *77*, 1173–1178, doi:10.1902/jop.2006.050233.

57. Heitmann, B.L.; Gamborg, M. Remaining Teeth, Cardiovascular Morbidity and Death among Adult Danes. *Prev. Med.* **2008**, *47*, 156–160, doi:10.1016/j.ypmed.2008.04.007.

58. Choe, H.; Kim, Y.H.; Park, J.W.; Kim, S.Y.; Lee, S.-Y.; Jee, S.H. Tooth Loss, Hypertension and Risk for Stroke in a Korean Population. *Atherosclerosis* **2009**, *203*, 550–556, doi:10.1016/j.atherosclerosis.2008.07.017.

59. Liljestrand, J.M.; Havulinna, A.S.; Paju, S.; Männistö, S.; Salomaa, V.; Pussinen, P.J. Missing Teeth Predict Incident Cardiovascular Events, Diabetes, and Death. *J. Dent. Res.* **2015**, *94*, 1055–1062, doi:10.1177/0022034515586352.

60. Chen, D.-Y.; Lin, C.-H.; Chen, Y.-M.; Chen, H.-H. Risk of Atrial Fibrillation or Flutter Associated with Periodontitis: A Nationwide, Population-Based, Cohort Study. *PloS One* **2016**, *11*, e0165601, doi:10.1371/journal.pone.0165601.

61. Leelaviwat, N.; Kewcharoen, J.; Poomprakobsri, K.; Trongtorsak, A.; Del Rio-Pertuz, G.; Abdelnabi, M.; Benjanuwattra, J.; Navaravong, L. Periodontal Disease and Risk of Atrial Fibrillation or Atrial Flutter: A Systematic Review and Meta-Analysis. *J. Arrhythmia* **2023**, *39*, 992–996, doi:10.1002/joa3.12921.

62. Zhang, Z.; Chen, F.; Gao, X.; Xiao, B.; Liu, F.; Lu, J. Effects of Oral Inflammatory Diseases and Oral Hygiene on Atrial Fibrillation: A Systematic Review. *Int. J. Clin. Pract.* **2023**, *2023*, 1750981, doi:10.1155/2023/1750981.

63. Leelapatana, P.; Limpuangthip, N. Association between Oral Health and Atrial Fibrillation: A Systematic Review. *Heliyon* **2022**, *8*, e09161, doi:10.1016/j.heliyon.2022.e09161.

64. LaMonte, M.J.; Genco, R.J.; Hovey, K.M.; Wallace, R.B.; Freudenheim, J.L.; Michaud, D.S.; Mai, X.; Tinker, L.F.; Salazar, C.R.; Andrews, C.A.; et al. History of Periodontitis Diagnosis and Edentulism as Predictors of Cardiovascular Disease, Stroke, and Mortality in Postmenopausal Women. *J. Am. Heart Assoc.* **2017**, *6*, e004518, doi:10.1161/JAHA.116.004518.

65. Cueto, A.; Mesa, F.; Bravo, M.; Ocaña-Riola, R. Periodontitis as Risk Factor for Acute Myocardial Infarction. A Case Control Study of Spanish Adults. *J. Periodontal Res.* **2005**, *40*, 36–42, doi:10.1111/j.1600-0765.2004.00766.x.

66. Shi, Q.; Zhang, B.; Huo, N.; Cai, C.; Liu, H.; Xu, J. Association between Myocardial Infarction and Periodontitis: A Meta-Analysis of Case-Control Studies. *Front. Physiol.* **2016**, *7*, 519, doi:10.3389/10yF-7016.00519.

67. Andriankaja, O.M.; Genco, R.J.; Dorn, J.; Dmochowski, J.; Hovey, K.; Falkner, K.L.; Trevisan, M. Periodontal Disease and Risk of Myocardial Infarction: The Role of Gender and Smoking. *Eur. J. Epidemiol.* **2007**, *22*, 699–705, doi:10.1007/s10654-007-9166-6.

68. Mucci, L.A.; Hsieh, C.-C.; Williams, P.L.; Arora, M.; Adami, H.-O.; de Faire, U.; Douglass, C.W.; Pedersen, N.L. Do Genetic Factors Explain the Association between Poor Oral Health and Cardiovascular Disease? A Prospective Study among Swedish Twins. *Am. J. Epidemiol.* **2009**, *170*, 615–621, doi:10.1093/aje/kwp177.

69. Dorn, J.M.; Genco, R.J.; Grossi, S.G.; Falkner, K.L.; Hovey, K.M.; Iacoviello, L.; Trevisan, M. Periodontal Disease and Recurrent Cardiovascular Events in Survivors of Myocardial Infarction (MI): The Western New York Acute MI Study. *J. Periodontol.* **2010**, *81*, 502–511, doi:10.1902/jop.2009.090499.

70. Lee, Y.-L.; Hu, H.-Y.; Chou, P.; Chu, D. Dental Prophylaxis Decreases the Risk of Acute Myocardial Infarction: A Nationwide Population-Based Study in Taiwan. *Clin. Interv. Aging* **2015**, *10*, 175–182, doi:10.2147/CIA.S67854.

71. Lee, J.-H.; Oh, J.-Y.; Youk, T.-M.; Jeong, S.-N.; Kim, Y.-T.; Choi, S.-H. Association between Periodontal Disease and Non-Communicable Diseases: A 12-Year Longitudinal Health-Examinee Cohort Study in South Korea. *Medicine (Baltimore)* **2017**, *96*, e7398, doi:10.1097/MD.0000000000007398.

72. Batty, G.D.; Jung, K.J.; Mok, Y.; Lee, S.J.; Back, J.H.; Lee, S.; Jee, S.H. Oral Health and Later Coronary Heart Disease: Cohort Study of One Million People. *Eur. J. Prev. Cardiol.* **2018**, *25*, 598–605, doi:10.1177/2047487318759112.

73. Mendez, M.V.; Scott, T.; LaMorte, W.; Vokonas, P.; Menzoian, J.O.; Garcia, R. An Association between Periodontal Disease and Peripheral Vascular Disease. *Am. J. Surg.* **1998**, *176*, 153–157, doi:10.1016/s0002-9610(98)00158-5.

74. Yang, S.; Zhao, L.S.; Cai, C.; Shi, Q.; Wen, N.; Xu, J. Association between Periodontitis and Peripheral Artery Disease: A Systematic Review and Meta-Analysis. *BMC Cardiovasc. Disord.* **2018**, *18*, 141, doi:10.1186/s12872-018-0879-0.

75. Loesche, W.J.; Schork, A.; Terpenning, M.S.; Chen, Y.M.; Dominguez, B.L.; Grossman, N. Assessing the Relationship between Dental Disease and Coronary Heart Disease in Elderly U.S. Veterans. *J. Am. Dent. Assoc. 1939* **1998**, *129*, 301–311, doi:10.14219/jada.archive.1998.0204.

76. Wu, T.; Trevisan, M.; Genco, R.; et al. Periodontal Disease as a Risk Factor for CVD, CHD, and Stroke. *Circulation* **1999**, *99*, 1109–1125.

77. Hujoel, P.P.; Drangsholt, M.; Spiekerman, C.; Derouen, T.A. Examining the Link between Coronary Heart Disease and the Elimination of Chronic Dental Infections. *J. Am. Dent. Assoc. 1939* **2001**, *132*, 883–889, doi:10.14219/jada.archive.2001.0300.

78. Rutger Persson, G.; Ohlsson, O.; Pettersson, T.; Renvert, S. Chronic Periodontitis, a Significant Relationship with Acute Myocardial Infarction. *Eur. Heart J.* **2003**, *24*, 2108–2115, doi:10.1016/j.ehj.2003.10.007.

79. Cabrera, C.; Hakeberg, M.; Ahlqwist, M.; Wedel, H.; Björkelund, C.; Bengtsson, C.; Lissner, L. Can the Relation between Tooth Loss and Chronic Disease Be Explained by Socio-Economic Status? A 24-Year Follow-up from the Population Study of Women in Gothenburg, Sweden. *Eur. J. Epidemiol.* **2005**, *20*, 229–236, doi:10.1007/s10654-004-5961-5.

80. Andriankaja, O.M.; Genco, R.J.; Dorn, J.; Dmochowski, J.; Hovey, K.; Falkner, K.L.; Scannapieco, F.; Trevisan, M. The Use of Different Measurements and Definitions of Periodontal Disease in the Study of the Association between Periodontal Disease and Risk of Myocardial Infarction. *J. Periodontol.* **2006**, *77*, 1067–1073, doi:10.1902/jop.2006.050276.

81. Lee, Y.-L.; Hu, H.-Y.; Huang, N.; Hwang, D.-K.; Chou, P.; Chu, D. Dental Prophylaxis and Periodontal Treatment Are Protective Factors to Ischemic Stroke. *Stroke* **2013**, *44*, 1026–1030, doi:10.1161/STROKEAHA.111.000076.

82. Lee, J.-H.; Lee, J.-S.; Park, J.-Y.; Choi, J.-K.; Kim, D.-W.; Kim, Y.-T.; Choi, S.-H. Association of Lifestyle-Related Comorbidities With Periodontitis: A Nationwide Cohort Study in Korea. *Medicine (Baltimore)* **2015**, *94*, e1567, doi:10.1097/MD.0000000000001567.

83. Sen, S.; Giamberardino, L.D.; Moss, K.; Morelli, T.; Rosamond, W.D.; Gottesman, R.F.; Beck, J.; Offenbacher, S. Periodontal Disease, Regular Dental Care Use, and Incident Ischemic Stroke. *Stroke* **2018**, *49*, 355–362, doi:10.1161/STROKEAHA.117.018990.

84. Lee, H.J.; Choi, E.K.; Park, J.B.; Han, K.D.; Oh, S. Tooth Loss Predicts Myocardial Infarction, Heart Failure, Stroke, and Death. *J. Dent. Res.* **2019**, *98*, 164–170, doi:10.1177/0022034518814829.

85. Mattila, K.J.; Valtonen, V.V.; Nieminen, M.; Huttunen, J.K. Dental Infection and the Risk of New Coronary Events: Prospective Study of Patients with Documented Coronary Artery Disease. *Clin. Infect. Dis. Off. Publ. Infect. Dis. Soc. Am.* **1995**, *20*, 588–592, doi:10.1093/clinids/20.3.588.

86. Emingil, G.; Buduneli, E.; Aliyev, A.; Akilli, A.; Atilla, G. Association between Periodontal Disease and Acute Myocardial Infarction. *J. Periodontol.* **2000**, *71*, 1882–1886, doi:10.1902/jop.2000.71.12.1882.

87. Beck, J.D.; Elter, J.R.; Heiss, G.; Couper, D.; Mauriello, S.M.; Offenbacher, S. Relationship of Periodontal Disease to Carotid Artery Intima-Media Wall Thickness: The Atherosclerosis Risk in Communities (ARIC) Study. *Arterioscler. Thromb. Vasc. Biol.* **2001**, *21*, 1816–1822, doi:10.1161/hq1101.097803.

88. Zeng, X.; Leng, W.; Lam, Y.; Yan, B.; Wei, X.; Weng, H.; Kwong, J. Periodontal Disease and Carotid Atherosclerosis: A Meta-Analysis of 17,330 Participants. *Int. J. Cardiol.* **2016**, *203*, 1044–1051, doi:10.1016/j.ijcard.2015.11.092.

89. Orlandi, M.; Suvan, J.; Petrie, A.; Donos, N.; Masi, S.; Hingorani, A.; Deanfield, J.; D’Aiuto, F. Association between Periodontal Disease and Its Treatment, Flow-Mediated Dilatation and Carotid Intima-Media Thickness: A Systematic Review and Meta-Analysis. *Atherosclerosis* **2014**, *236*, 39–46, doi:10.1016/j.atherosclerosis.2014.06.002.

90. Katz, J.; Chaushu, G.; Sharabi, Y. On the Association between Hypercholesterolemia, Cardiovascular Disease and Severe Periodontal Disease. *J. Clin. Periodontol.* **2001**, *28*, 865–868, doi:10.1034/j.1600-051x.2001.028009865.x.

91. Bloemenkamp, D.G.M.; van den Bosch, M.A.A.J.; Mali, W.P.T.M.; Tanis, B.C.; Rosendaal, F.R.; Kemmeren, J.M.; Algra, A.; Visseren, F.L.J.; van der Graaf, Y. Novel Risk Factors for Peripheral Arterial Disease in Young Women. *Am. J. Med.* **2002**, *113*, 462–467, doi:10.1016/s0002-9343(02)01258-5.

92. Persson, R.E.; Hollender, L.G.; Powell, V.L.; MacEntee, M.; Wyatt, C.C.L.; Kiyak, H.A.; Persson, G.R. Assessment of Periodontal Conditions and Systemic Disease in Older Subjects. II. Focus on Cardiovascular Diseases. *J. Clin. Periodontol.* **2002**, *29*, 803–810, doi:10.1034/j.1600-051x.2002.290903.x.

93. Ravon, N.A.; Hollender, L.G.; McDonald, V.; Persson, G.R. Signs of Carotid Calcification from Dental Panoramic Radiographs Are in Agreement with Doppler Sonography Results. *J. Clin. Periodontol.* **2003**, *30*, 1084–1090, doi:10.1046/j.0303-6979.2003.00427.x.

94. Wang, W.; Yang, Z.; Wang, Y.; Gao, H.; Wang, Y.; Zhang, Q. Association between Periodontitis and Carotid Artery Calcification: A Systematic Review and Meta-Analysis. *BioMed Res. Int.* **2021**, *2021*, 3278351, doi:10.1155/2021/3278351.

95. Ajwani, S.; Mattila, K.J.; Tilvis, R.S.; Ainamo, A. Periodontal Disease and Mortality in an Aged Population. *Spec. Care Dent. Off. Publ. Am. Assoc. Hosp. Dent. Acad. Dent. Handicap. Am. Soc. Geriatr. Dent.* **2003**, *23*, 125–130, doi:10.1111/j.1754-4505.2003.tb00297.x.

96. Dörfer, C.E.; Becher, H.; Ziegler, C.M.; Kaiser, C.; Lutz, R.; Jörss, D.; Lichy, C.; Buggle, F.; Bültmann, S.; Preusch, M.; et al. The Association of Gingivitis and Periodontitis with Ischemic Stroke. *J. Clin. Periodontol.* **2004**, *31*, 396–401, doi:10.1111/j.1600-051x.2004.00579.x.

97. Grau, A.J.; Becher, H.; Ziegler, C.M.; Lichy, C.; Buggle, F.; Kaiser, C.; Lutz, R.; Bültmann, S.; Preusch, M.; Dörfer, C.E. Periodontal Disease as a Risk Factor for Ischemic Stroke. *Stroke* **2004**, *35*, 496–501, doi:10.1161/01.STR.0000110789.20526.9D.

98. Geerts, S.O.; Legrand, V.; Charpentier, J.; Albert, A.; Rompen, E.H. Further Evidence of the Association between Periodontal Conditions and Coronary Artery Disease. *J. Periodontol.* **2004**, *75*, 1274–1280, doi:10.1902/jop.2004.75.9.1274.

99. Elter, J.R.; Champagne, C.M.E.; Offenbacher, S.; Beck, J.D. Relationship of Periodontal Disease and Tooth Loss to Prevalence of Coronary Heart Disease. *J. Periodontol.* **2004**, *75*, 782–790, doi:10.1902/jop.2004.75.6.782.

100. Söder, P.-O.; Söder, B.; Nowak, J.; Jogestrand, T. Early Carotid Atherosclerosis in Subjects with Periodontal Diseases. *Stroke* **2005**, *36*, 1195–1200, doi:10.1161/01.STR.0000165916.90593.cb.

101. Buhlin, K.; Gustafsson, A.; Ahnve, S.; Janszky, I.; Tabrizi, F.; Klinge, B. Oral Health in Women with Coronary Heart Disease. *J. Periodontol.* **2005**, *76*, 544–550, doi:10.1902/jop.2005.76.4.544.

102. Briggs, J.E.; McKeown, P.P.; Crawford, V.L.S.; Woodside, J.V.; Stout, R.W.; Evans, A.; Linden, G.J. Angiographically Confirmed Coronary Heart Disease and Periodontal Disease in Middle-Aged Males. *J. Periodontol.* **2006**, *77*, 95–102, doi:10.1902/jop.2006.77.1.95.

103. Padilha, D.M.P.; Hilgert, J.B.; Hugo, F.N.; Bós, A.J.G.; Ferrucci, L. Number of Teeth and Mortality Risk in the Baltimore Longitudinal Study of Aging. *J. Gerontol. A. Biol. Sci. Med. Sci.* **2008**, *63*, 739–744, doi:10.1093/gerona/63.7.739.

104. Osterberg, T.; Carlsson, G.E.; Sundh, V.; Mellström, D. Number of Teeth--a Predictor of Mortality in 70-Year-Old Subjects. *Community Dent. Oral Epidemiol.* **2008**, *36*, 258–268, doi:10.1111/j.1600-0528.2007.00413.x.

105. Chen, Y.-W.; Umeda, M.; Nagasawa, T.; Takeuchi, Y.; Huang, Y.; Inoue, Y.; Iwai, T.; Izumi, Y.; Ishikawa, I. Periodontitis May Increase the Risk of Peripheral Arterial Disease. *Eur. J. Vasc. Endovasc. Surg. Off. J. Eur. Soc. Vasc. Surg.* **2008**, *35*, 153–158, doi:10.1016/j.ejvs.2007.08.016.

106. Sim, S.-J.; Kim, H.-D.; Moon, J.-Y.; Zavras, A.I.; Zdanowicz, J.; Jang, S.-J.; Jin, B.-H.; Bae, K.-H.; Paik, D.-I.; Douglass, C.W. Periodontitis and the Risk for Non-Fatal Stroke in Korean Adults. *J. Periodontol.* **2008**, *79*, 1652–1658, doi:10.1902/jop.2008.080015.

107. Pradeep, A.R.; Hadge, P.; Arjun Raju, P.; Shetty, S.R.; Shareef, K.; Guruprasad, C.N. Periodontitis as a Risk Factor for Cerebrovascular Accident: A Case-Control Study in the Indian Population. *J. Periodontal Res.* **2010**, *45*, 223–228, doi:10.1111/j.1600-0765.2009.01220.x.

108. Holmlund, A.; Hedin, M.; Pussinen, P.J.; Lerner, U.H.; Lind, L. Porphyromonas Gingivalis (Pg) a Possible Link between Impaired Oral Health and Acute Myocardial Infarction. *Int. J. Cardiol.* **2011**, *148*, 148–153, doi:10.1016/j.ijcard.2009.10.034.

109. Vieira, C.L.Z.; Cury, P.R.; Miname, M.H.; Martinez, L.R.; Bortolotto, L.A.; Giuliano, I.B.; Santos, R.D.; Caramelli, B. Severe Periodontitis Is Associated with Diastolic Blood Pressure Elevation in Individuals with Heterozygous Familial Hypercholesterolemia: A Pilot Study. *J. Periodontol.* **2011**, *82*, 683–688, doi:10.1902/jop.2010.100496.

110. Schmitt, A.; Carra, M.C.; Boutouyrie, P.; Bouchard, P. Periodontitis and Arterial Stiffness: A Systematic Review and Meta-Analysis. *J. Clin. Periodontol.* **2015**, *42*, 977–987, doi:10.1111/jcpe.12467.

111. López-Jornet, P.; Berná-Mestre, J.D.; Berná-Serna, J.D.; Camacho-Alonso, F.; Fernandez-Millan, S.; Reus-Pintado, M. Measurement of Atherosclerosis Markers in Patients with Periodontitis: A Case-Control Study. *J. Periodontol.* **2012**, *83*, 690–698, doi:10.1902/jop.2011.110412.

112. Schwahn, C.; Polzer, I.; Haring, R.; Dörr, M.; Wallaschofski, H.; Kocher, T.; Mundt, T.; Holtfreter, B.; Samietz, S.; Völzke, H.; et al. Missing, Unreplaced Teeth and Risk of All-Cause and Cardiovascular Mortality. *Int. J. Cardiol.* **2013**, *167*, 1430–1437, doi:10.1016/j.ijcard.2012.04.061.

113. Soto-Barreras, U.; Olvera-Rubio, J.O.; Loyola-Rodriguez, J.P.; Reyes-Macias, J.F.; Martinez-Martinez, R.E.; Patiño-Marin, N.; Martinez-Castañon, G.A.; Aradillas-Garcia, C.; Little, J.W. Peripheral Arterial Disease Associated with Caries and Periodontal Disease. *J. Periodontol.* **2013**, *84*, 486–494, doi:10.1902/jop.2012.120051.

114. Khosravi Samani, M.; Jalali, F.; Seyyed Ahadi, S.M.; Hoseini, S.R.; Dabbagh Sattari, F. The Relationship between Acute Myocardial Infarction and Periodontitis. *Casp. J. Intern. Med.* **2013**, *4*, 667–671.

115. Wożakowska-Kapłon, B.; Włosowicz, M.; Gorczyca-Michta, I.; Górska, R. Oral Health Status and the Occurrence and Clinical Course of Myocardial Infarction in Hospital Phase: A Case-Control Study. *Cardiol. J.* **2013**, *20*, 370–377, doi:10.5603/CJ.2013.0095.

116. Hayashida, H.; Saito, T.; Kawasaki, K.; Kitamura, M.; Furugen, R.; Iwasaki, T.; Hayashida, Y.; Nakazato, M.; Sekita, T.; Takamura, N.; et al. Association of Periodontitis with Carotid Artery Intima-Media Thickness and Arterial Stiffness in Community-Dwelling People in Japan: The Nagasaki Islands Study. *Atherosclerosis* **2013**, *229*, 186–191, doi:10.1016/j.atherosclerosis.2013.04.002.

117. Pinho, M.M.; Faria-Almeida, R.; Azevedo, E.; Manso, M.C.; Martins, L. Periodontitis and Atherosclerosis: An Observational Study. *J. Periodontal Res.* **2013**, *48*, 452–457, doi:10.1111/jre.12026.

118. Iwashima, Y.; Kokubo, Y.; Ono, T.; Yoshimuta, Y.; Kida, M.; Kosaka, T.; Maeda, Y.; Kawano, Y.; Miyamoto, Y. Additive Interaction of Oral Health Disorders on Risk of Hypertension in a Japanese Urban Population: The Suita Study. *Am. J. Hypertens.* **2014**, *27*, 710–719, doi:10.1093/ajh/hpt227.

119. Jung, Y.-S.; Shin, M.-H.; Kim, I.-S.; Kweon, S.-S.; Lee, Y.-H.; Kim, O.-J.; Kim, Y.-J.; Chung, H.-J.; Kim, O.-S. Relationship between Periodontal Disease and Subclinical Atherosclerosis: The Dong-Gu Study. *J. Clin. Periodontol.* **2014**, *41*, 262–268, doi:10.1111/jcpe.12204.

120. Willershausen, I.; Weyer, V.; Peter, M.; Weichert, C.; Kasaj, A.; Münzel, T.; Willershausen, B. Association between Chronic Periodontal and Apical Inflammation and Acute Myocardial Infarction. *Odontology* **2014**, *102*, 297–302, doi:10.1007/s10266-013-0112-7.

121. Kodovazenitis, G.; Pitsavos, C.; Papadimitriou, L.; Vrotsos, I.A.; Stefanadis, C.; Madianos, P.N. Association between Periodontitis and Acute Myocardial Infarction: A Case-Control Study of a Nondiabetic Population. *J. Periodontal Res.* **2014**, *49*, 246–252, doi:10.1111/jre.12101.

122. Chou, S.-H.; Tung, Y.-C.; Lin, Y.-S.; Wu, L.-S.; Lin, C.-P.; Liou, E.J.-W.; Chang, C.-J.; Kung, S.; Chu, P.-H. Major Adverse Cardiovascular Events in Treated Periodontitis: A Population-Based Follow-Up Study from Taiwan. *PloS One* **2015**, *10*, e0130807, doi:10.1371/journal.pone.0130807.

123. Darnaud, C.; Thomas, F.; Pannier, B.; Danchin, N.; Bouchard, P. Oral Health and Blood Pressure: The IPC Cohort. *Am. J. Hypertens.* **2015**, *28*, 1257–1261, doi:10.1093/ajh/hpv025.

124. Ahn, Y.-B.; Shin, M.-S.; Byun, J.-S.; Kim, H.-D. The Association of Hypertension with Periodontitis Is Highlighted in Female Adults: Results from the Fourth Korea National Health and Nutrition Examination Survey. *J. Clin. Periodontol.* **2015**, *42*, 998–1005, doi:10.1111/jcpe.12471.

125. Vedin, O.; Hagström, E.; Budaj, A.; Denchev, S.; Harrington, R.A.; Koenig, W.; Soffer, J.; Sritara, P.; Stebbins, A.; Stewart, R.H.; et al. Tooth Loss Is Independently Associated with Poor Outcomes in Stable Coronary Heart Disease. *Eur. J. Prev. Cardiol.* **2016**, *23*, 839–846, doi:10.1177/2047487315621978.

126. Ahn, Y.-B.; Shin, M.-S.; Han, D.-H.; Sukhbaatar, M.; Kim, M.-S.; Shin, H.-S.; Kim, H.-D. Periodontitis Is Associated with the Risk of Subclinical Atherosclerosis and Peripheral Arterial Disease in Korean Adults. *Atherosclerosis* **2016**, *251*, 311–318, doi:10.1016/j.atherosclerosis.2016.07.898.

127. Rydén, L.; Buhlin, K.; Ekstrand, E.; de Faire, U.; Gustafsson, A.; Holmer, J.; Kjellström, B.; Lindahl, B.; Norhammar, A.; Nygren, Å.; et al. Periodontitis Increases the Risk of a First Myocardial Infarction: A Report From the PAROKRANK Study. *Circulation* **2016**, *133*, 576–583, doi:10.1161/CIRCULATIONAHA.115.020324.

128. Çalapkorur, M.U.; Alkan, B.A.; Tasdemir, Z.; Akcali, Y.; Saatçi, E. Association of Peripheral Arterial Disease with Periodontal Disease: Analysis of Inflammatory Cytokines and an Acute Phase Protein in Gingival Crevicular Fluid and Serum. *J. Periodontal Res.* **2017**, *52*, 532–539, doi:10.1111/jre.12419.

129. Lin, H.-W.; Chen, C.-M.; Yeh, Y.-C.; Chen, Y.-Y.; Guo, R.-Y.; Lin, Y.-P.; Li, Y.-C. Dental Treatment Procedures for Periodontal Disease and the Subsequent Risk of Ischaemic Stroke: A Retrospective Population-Based Cohort Study. *J. Clin. Periodontol.* **2019**, *46*, 642–649, doi:10.1111/jcpe.13113.

130. Chang, Y.; Woo, H.G.; Park, J.; Lee, J.S.; Song, T.-J. Improved Oral Hygiene Care Is Associated with Decreased Risk of Occurrence for Atrial Fibrillation and Heart Failure: A Nationwide Population-Based Cohort Study. *Eur. J. Prev. Cardiol.* **2020**, *27*, 1835–1845, doi:10.1177/2047487319886018.

131. Sen, S.; Redd, K.; Trivedi, T.; Moss, K.; Alonso, A.; Soliman, E.Z.; Magnani, J.W.; Chen, L.Y.; Gottesman, R.F.; Rosamond, W.; et al. Periodontal Disease, Atrial Fibrillation and Stroke. *Am. Heart J.* **2021**, *235*, 36–43, doi:10.1016/j.ahj.2021.01.009.

132. Shimazaki, Y.; Soh, I.; Saito, T.; Yamashita, Y.; Koga, T.; Miyazaki, H.; Takehara, T. Influence of Dentition Status on Physical Disability, Mental Impairment, and Mortality in Institutionalized Elderly People. *J. Dent. Res.* **2001**, *80*, 340–345, doi:10.1177/00220345010800010801.

133. Jansson, L.; Lavstedt, S.; Frithiof, L.; Theobald, H. Relationship between Oral Health and Mortality in Cardiovascular Diseases. *J. Clin. Periodontol.* **2001**, *28*, 762–768, doi:10.1034/j.1600-051x.2001.280807.x.

134. Bazile, A.; Bissada, N.F.; Nair, R.; Siegel, B.P. Periodontal Assessment of Patients Undergoing Angioplasty for Treatment of Coronary Artery Disease. *J. Periodontol.* **2002**, *73*, 631–636, doi:10.1902/jop.2002.73.6.631.

135. Malthaner, S.C.; Moore, S.; Mills, M.; Saad, R.; Sabatini, R.; Takacs, V.; McMahan, A.C.; Oates, T.W. Investigation of the Association between Angiographically Defined Coronary Artery Disease and Periodontal Disease. *J. Periodontol.* **2002**, *73*, 1169–1176, doi:10.1902/jop.2002.73.10.1169.

136. López, R.; Oyarzún, M.; Naranjo, C.; Cumsille, F.; Ortiz, M.; Baelum, V. Coronary Heart Disease and Periodontitis -- a Case Control Study in Chilean Adults. *J. Clin. Periodontol.* **2002**, *29*, 468–473, doi:10.1034/j.1600-051x.2002.290513.x.

137. Buhlin, K.; Gustafsson, A.; Pockley, A.G.; Frostegård, J.; Klinge, B. Risk Factors for Cardiovascular Disease in Patients with Periodontitis. *Eur. Heart J.* **2003**, *24*, 2099–2107, doi:10.1016/j.ehj.2003.09.016.

138. Hämäläinen, P.; Meurman, J.H.; Keskinen, M.; Heikkinen, E. Relationship between Dental Health and 10-Year Mortality in a Cohort of Community-Dwelling Elderly People. *Eur. J. Oral Sci.* **2003**, *111*, 291–296, doi:10.1034/j.1600-0722.2003.00055.x.

139. Elter, J.R.; Offenbacher, S.; Toole, J.F.; Beck, J.D. Relationship of Periodontal Disease and Edentulism to Stroke/TIA. *J. Dent. Res.* **2003**, *82*, 998–1001, doi:10.1177/154405910308201212.

140. Buhlin, K.; Gustafsson, A.; Håkansson, J.; Klinge, B. Self-Reported Oral Health, Dental Care Habits and Cardiovascular Disease in an Adult Swedish Population. *Oral Health Prev. Dent.* **2003**, *1*, 291–299.

141. Desvarieux, M.; Schwahn, C.; Völzke, H.; Demmer, R.T.; Lüdemann, J.; Kessler, C.; Jacobs, D.R.; John, U.; Kocher, T. Gender Differences in the Relationship between Periodontal Disease, Tooth Loss, and Atherosclerosis. *Stroke* **2004**, *35*, 2029–2035, doi:10.1161/01.STR.0000136767.71518.36.

142. Ragnarsson, E.; Eliasson, S.T.; Gudnason, V. Loss of Teeth and Coronary Heart Disease. *Int. J. Prosthodont.* **2004**, *17*, 441–446.

143. Renvert, S.; Ohlsson, O.; Persson, S.; Lang, N.P.; Persson, G.R. Analysis of Periodontal Risk Profiles in Adults with or without a History of Myocardial Infarction. *J. Clin. Periodontol.* **2004**, *31*, 19–24, doi:10.1111/j.0303-6979.2004.00431.x.

144. Montebugnoli, L.; Servidio, D.; Miaton, R.A.; Prati, C.; Tricoci, P.; Melloni, C. Poor Oral Health Is Associated with Coronary Heart Disease and Elevated Systemic Inflammatory and Haemostatic Factors. *J. Clin. Periodontol.* **2004**, *31*, 25–29, doi:10.1111/j.0303-6979.2004.00432.x.

145. Engebretson, S.P.; Lamster, I.B.; Elkind, M.S.V.; Rundek, T.; Serman, N.J.; Demmer, R.T.; Sacco, R.L.; Papapanou, P.N.; Desvarieux, M. Radiographic Measures of Chronic Periodontitis and Carotid Artery Plaque. *Stroke J. Cereb. Circ.* **2005**, *36*, 561–566, doi:10.1161/01.STR.0000155734.34652.6c.

146. Leivadaros, E.; van der Velden, U.; Bizzarro, S.; ten Heggeler, J.M.A.G.; Gerdes, V.E.A.; Hoek, F.J.; Nagy, T.O.M.; Scholma, J.; Bakker, S.J.L.; Gans, R.O.B.; et al. A Pilot Study into Measurements of Markers of Atherosclerosis in Periodontitis. *J. Periodontol.* **2005**, *76*, 121–128, doi:10.1902/jop.2005.76.1.121.

147. Beck, J.D.; Eke, P.; Lin, D.; Madianos, P.; Couper, D.; Moss, K.; Elter, J.; Heiss, G.; Offenbacher, S. Associations between IgG Antibody to Oral Organisms and Carotid Intima-Medial Thickness in Community-Dwelling Adults. *Atherosclerosis* **2005**, *183*, 342–348, doi:10.1016/j.atherosclerosis.2005.03.017.

148. Desvarieux, M.; Demmer, R.T.; Rundek, T.; Boden-Albala, B.; Jacobs, D.R.; Sacco, R.L.; Papapanou, P.N. Periodontal Microbiota and Carotid Intima-Media Thickness: The Oral Infections and Vascular Disease Epidemiology Study (INVEST). *Circulation* **2005**, *111*, 576–582, doi:10.1161/01.CIR.0000154582.37101.15.

149. Seinost, G.; Wimmer, G.; Skerget, M.; Thaller, E.; Brodmann, M.; Gasser, R.; Bratschko, R.O.; Pilger, E. Periodontal Treatment Improves Endothelial Dysfunction in Patients with Severe Periodontitis. *Am. Heart J.* **2005**, *149*, 1050–1054, doi:10.1016/j.ahj.2004.09.059.

150. Hämäläinen, P.; Meurman, J.H.; Kauppinen, M.; Keskinen, M. Oral Infections as Predictors of Mortality. *Gerodontology* **2005**, *22*, 151–157, doi:10.1111/j.1741-2358.2005.00064.x.

151. Coelho, J.M.F.; Gomes Filho, I.S.; Santos, C.A. de S.T.; Marques Neto, J.; Viana, M.I.P.; Cruz, S.S. da; Sarmento, V.A. Doença periodontal e doença cardiovascular: um estudo piloto. *Rev Baiana Saúde Pública* **2005**, 251–261.

152. Völzke, H.; Schwahn, C.; Dörr, M.; Schwarz, S.; Robinson, D.; Dören, M.; Rettig, R.; Felix, S.B.; John, U.; Kocher, T. Gender Differences in the Relation between Number of Teeth and Systolic Blood Pressure. *J. Hypertens.* **2006**, *24*, 1257–1263, doi:10.1097/01.hjh.0000234104.15992.df.

153. Schillinger, T.; Kluger, W.; Exner, M.; Mlekusch, W.; Sabeti, S.; Amighi, J.; Wagner, O.; Minar, E.; Schillinger, M. Dental and Periodontal Status and Risk for Progression of Carotid Atherosclerosis: The Inflammation and Carotid Artery Risk for Atherosclerosis Study Dental Substudy. *Stroke* **2006**, *37*, 2271–2276, doi:10.1161/01.STR.0000236495.82545.2e.

154. Morita, I.; Nakagaki, H.; Kato, K.; Murakami, T.; Tsuboi, S.; Hayashizaki, J.; Toyama, A.; Hashimoto, M.; Simozato, T.; Morishita, N.; et al. Relationship between Survival Rates and Numbers of Natural Teeth in an Elderly Japanese Population. *Gerodontology* **2006**, *23*, 214–218, doi:10.1111/j.1741-2358.2006.00134.x.

155. Geismar, K.; Stoltze, K.; Sigurd, B.; Gyntelberg, F.; Holmstrup, P. Periodontal Disease and Coronary Heart Disease. *J. Periodontol.* **2006**, *77*, 1547–1554, doi:10.1902/jop.2006.050405.

156. Spahr, A.; Klein, E.; Khuseyinova, N.; Boeckh, C.; Muche, R.; Kunze, M.; Rothenbacher, D.; Pezeshki, G.; Hoffmeister, A.; Koenig, W. Periodontal Infections and Coronary Heart Disease: Role of Periodontal Bacteria and Importance of Total Pathogen Burden in the Coronary Event and Periodontal Disease (CORODONT) Study. *Arch. Intern. Med.* **2006**, *166*, 554–559, doi:10.1001/archinte.166.5.554.

157. Engström, S.; Gahnberg, L.; Högberg, H.; Svärdsudd, K. Association between High Blood Pressure and Deep Periodontal Pockets: A Nested Case-Referent Study. *Ups. J. Med. Sci.* **2007**, *112*, 95–103, doi:10.3109/2000-1967-099.

158. Tonetti, M.S.; D’Aiuto, F.; Nibali, L.; Donald, A.; Storry, C.; Parkar, M.; Suvan, J.; Hingorani, A.D.; Vallance, P.; Deanfield, J. Treatment of Periodontitis and Endothelial Function. *N. Engl. J. Med.* **2007**, *356*, 911–920, doi:10.1056/NEJMoa063186.

159. Osterberg, T.; Carlsson, G.E.; Sundh, V.; Steen, B. Number of Teeth--a Predictor of Mortality in the Elderly? A Population Study in Three Nordic Localities. *Acta Odontol. Scand.* **2007**, *65*, 335–340, doi:10.1080/00016350701739519.

160. Nonnenmacher, C.; Stelzel, M.; Susin, C.; Sattler, A.M.; Schaefer, J.R.; Maisch, B.; Mutters, R.; Flores-de-Jacoby, L. Periodontal Microbiota in Patients with Coronary Artery Disease Measured by Real-Time Polymerase Chain Reaction: A Case-Control Study. *J. Periodontol.* **2007**, *78*, 1724–1730, doi:10.1902/jop.2007.060345.

161. Rech, R.L.; Nurkin, N.; da Cruz, I.; Sostizzo, F.; Baião, C.; Perrone, J.A.; Wainstein, R.; Pretto, D.; Manenti, E.R.F.; Bodanese, L.C. Association between Periodontal Disease and Acute Coronary Syndrome. *Arq. Bras. Cardiol.* **2007**, *88*, 185–190, doi:10.1590/s0066-782x2007000200009.

162. Senba, T.; Kobayashi, Y.; Inoue, K.; Kaneto, C.; Inoue, M.; Toyokawa, S.; Suyama, Y.; Suzuki, T.; Miyano, Y.; Miyoshi, Y. The Association between Self-Reported Periodontitis and Coronary Heart Disease--from MY Health Up Study--. *J. Occup. Health* **2008**, *50*, 283–287, doi:10.1539/joh.l7066.

163. Higashi, Y.; Goto, C.; Jitsuiki, D.; Umemura, T.; Nishioka, K.; Hidaka, T.; Takemoto, H.; Nakamura, S.; Soga, J.; Chayama, K.; et al. Periodontal Infection Is Associated with Endothelial Dysfunction in Healthy Subjects and Hypertensive Patients. *Hypertens. Dallas Tex 1979* **2008**, *51*, 446–453, doi:10.1161/HYPERTENSIONAHA.107.101535.

164. Holm-Pedersen, P.; Schultz-Larsen, K.; Christiansen, N.; Avlund, K. Tooth Loss and Subsequent Disability and Mortality in Old Age. *J. Am. Geriatr. Soc.* **2008**, *56*, 429–435, doi:10.1111/j.1532-5415.2007.01602.x.

165. Fukai, K.; Takiguchi, T.; Ando, Y.; Aoyama, H.; Miyakawa, Y.; Ito, G.; Inoue, M.; Sasaki, H. Mortality Rates of Community-Residing Adults with and without Dentures. *Geriatr. Gerontol. Int.* **2008**, *8*, 152–159, doi:10.1111/j.1447-0594.2008.00464.x.

166. Franek, E.; Klamczynska, E.; Ganowicz, E.; Blach, A.; Budlewski, T.; Gorska, R. Association of Chronic Periodontitis with Left Ventricular Mass and Central Blood Pressure in Treated Patients with Essential Hypertension. *Am. J. Hypertens.* **2009**, *22*, 203–207, doi:10.1038/ajh.2008.330.

167. Brown, D.W. Complete Edentulism Prior to the Age of 65 Years Is Associated with All-Cause Mortality. *J. Public Health Dent.* **2009**, *69*, 260–266, doi:10.1111/j.1752-7325.2009.00132.x.

168. Higashi, Y.; Goto, C.; Hidaka, T.; Soga, J.; Nakamura, S.; Fujii, Y.; Hata, T.; Idei, N.; Fujimura, N.; Chayama, K.; et al. Oral Infection-Inflammatory Pathway, Periodontitis, Is a Risk Factor for Endothelial Dysfunction in Patients with Coronary Artery Disease. *Atherosclerosis* **2009**, *206*, 604–610, doi:10.1016/j.atherosclerosis.2009.03.037.

169. Carallo, C.; Fortunato, L.; de Franceschi, M.S.; Irace, C.; Tripolino, C.; Cristofaro, M.G.; Giudice, M.; Gnasso, A. Periodontal Disease and Carotid Atherosclerosis: Are Hemodynamic Forces a Link? *Atherosclerosis* **2010**, *213*, 263–267, doi:10.1016/j.atherosclerosis.2010.07.025.

170. Tsakos, G.; Sabbah, W.; Hingorani, A.D.; Netuveli, G.; Donos, N.; Watt, R.G.; D’Aiuto, F. Is Periodontal Inflammation Associated with Raised Blood Pressure? Evidence from a National US Survey. *J. Hypertens.* **2010**, *28*, 2386–2393, doi:10.1097/HJH.0b013e32833e0fe1.

171. Nesse, W.; Dijkstra, P.U.; Abbas, F.; Spijkervet, F.K.L.; Stijger, A.; Tromp, J.A.H.; van Dijk, J.L.; Vissink, A. Increased Prevalence of Cardiovascular and Autoimmune Diseases in Periodontitis Patients: A Cross-Sectional Study. *J. Periodontol.* **2010**, *81*, 1622–1628, doi:10.1902/jop.2010.100058.

172. Desvarieux, M.; Demmer, R.T.; Jacobs, D.R.; Rundek, T.; Boden-Albala, B.; Sacco, R.L.; Papapanou, P.N. Periodontal Bacteria and Hypertension: The Oral Infections and Vascular Disease Epidemiology Study (INVEST). *J. Hypertens.* **2010**, *28*, 1413–1421, doi:10.1097/HJH.0b013e328338cd36.

173. Islas-Granillo, H.; Borges-Yañez, S.A.; Lucas-Rincón, S.E.; Medina-Solís, C.E.; Casanova-Rosado, A.J.; Márquez-Corona, M.L.; Maupomé, G. Edentulism Risk Indicators among Mexican Elders 60-Year-Old and Older. *Arch. Gerontol. Geriatr.* **2011**, *53*, 258–262, doi:10.1016/j.archger.2010.12.014.

174. Paganini-Hill, A.; White, S.C.; Atchison, K.A. Dental Health Behaviors, Dentition, and Mortality in the Elderly: The Leisure World Cohort Study. *J. Aging Res.* **2011**, *2011*, 156061, doi:10.4061/2011/156061.

175. Yamori, M.; Njelekela, M.; Mtabaji, J.; Yamori, Y.; Bessho, K. Hypertension, Periodontal Disease, and Potassium Intake in Nonsmoking, Nondrinker African Women on No Medication. *Int. J. Hypertens.* **2011**, *2011*, 695719, doi:10.4061/2011/695719.

176. Vidal, F.; Figueredo, C.M.S.; Cordovil, I.; Fischer, R.G. Higher Prevalence of Periodontitis in Patients with Refractory Arterial Hypertension: A Case-Control Study. *Oral Dis.* **2011**, *17*, 560–563, doi:10.1111/j.1601-0825.2011.01800.x.

177. Watt, R.G.; Tsakos, G.; de Oliveira, C.; Hamer, M. Tooth Loss and Cardiovascular Disease Mortality Risk--Results from the Scottish Health Survey. *PloS One* **2012**, *7*, e30797, doi:10.1371/journal.pone.0030797.

178. Franek, E.; Januszkiewicz-Caulier, J.; Błach, A.; Napora, M.; Jedynasty, K.; Budlewski, T.; Gozdowski, D.; Górska, R. Intima-Media Thickness and Other Markers of Atherosclerosis in Patients with Type 2 Diabetes and Periodontal Disease. *Kardiol. Pol.* **2012**, *70*, 7–13.

179. Chen, S.-J.; Liu, C.-J.; Chao, T.-F.; Wang, K.-L.; Chen, T.-J.; Chou, P.; Wang, F.-D.; Lin, S.-J.; Chiang, C.-E. Dental Scaling and Atrial Fibrillation: A Nationwide Cohort Study. *Int. J. Cardiol.* **2013**, *168*, 2300–2303, doi:10.1016/j.ijcard.2013.01.192.

180. Hayasaka, K.; Tomata, Y.; Aida, J.; Watanabe, T.; Kakizaki, M.; Tsuji, I. Tooth Loss and Mortality in Elderly Japanese Adults: Effect of Oral Care. *J. Am. Geriatr. Soc.* **2013**, *61*, 815–820, doi:10.1111/jgs.12225.

181. Parkar, S.M.; Modi, G.N.; Jani, J. Periodontitis as Risk Factor for Acute Myocardial Infarction: A Case Control Study. *Heart Views Off. J. Gulf Heart Assoc.* **2013**, *14*, 5–11, doi:10.4103/1995-705X.107113.

182. Li, P.; He, L.; Sha, Y.; Luan, Q. [Periodontal status of patients with post-acute myocardial infarction]. *Beijing Da Xue Xue Bao* **2013**, *45*, 22–26.

183. Ramesh, A.; Soroushian, S.; Ganguly, R. Coincidence of Calcified Carotid Atheromatous Plaque, Osteoporosis, and Periodontal Bone Loss in Dental Panoramic Radiographs. *Imaging Sci. Dent.* **2013**, *43*, 235–243, doi:10.5624/isd.2013.43.4.235.

184. Rivas-Tumanyan, S.; Campos, M.; Zevallos, J.C.; Joshipura, K.J. Periodontal Disease, Hypertension, and Blood Pressure among Older Adults in Puerto Rico. *J. Periodontol.* **2013**, *84*, 203–211, doi:10.1902/jop.2012.110748.

185. Vidal, F.; Cordovil, I.; Figueredo, C.M.S.; Fischer, R.G. Non-Surgical Periodontal Treatment Reduces Cardiovascular Risk in Refractory Hypertensive Patients: A Pilot Study. *J. Clin. Periodontol.* **2013**, *40*, 681–687, doi:10.1111/jcpe.12110.

186. Janket, S.-J.; Baird, A.E.; Jones, J.A.; Jackson, E.A.; Surakka, M.; Tao, W.; Meurman, J.H.; Van Dyke, T.E. Number of Teeth, C-Reactive Protein, Fibrinogen and Cardiovascular Mortality: A 15-Year Follow-up Study in a Finnish Cohort. *J. Clin. Periodontol.* **2014**, *41*, 131–140, doi:10.1111/jcpe.12192.

187. Ando, A.; Tanno, K.; Ohsawa, M.; Onoda, T.; Sakata, K.; Tanaka, F.; Makita, S.; Nakamura, M.; Omama, S.; Ogasawara, K.; et al. Associations of Number of Teeth with Risks for All-Cause Mortality and Cause-Specific Mortality in Middle-Aged and Elderly Men in the Northern Part of Japan: The Iwate-KENCO Study. *Community Dent. Oral Epidemiol.* **2014**, *42*, 358–365, doi:10.1111/cdoe.12095.

188. Yu, H.; Qi, L.T.; Liu, L.S.; Wang, X.Y.; Zhang, Y.; Huo, Y.; Luan, Q.X. Association of Carotid Intima–Media Thickness and Atherosclerotic Plaque with Periodontal Status. *J. Dent. Res.* **2014**, *93*, 744–751, doi:10.1177/0022034514538973.

189. Machida, T.; Tomofuji, T.; Ekuni, D.; Azuma, T.; Takeuchi, N.; Maruyama, T.; Mizutani, S.; Kataoka, K.; Kawabata, Y.; Morita, M. Severe Periodontitis Is Inversely Associated with Coffee Consumption in the Maintenance Phase of Periodontal Treatment. *Nutrients* **2014**, *6*, 4476–4490, doi:10.3390/nu6104476.

190. Ollikainen, E.; Saxlin, T.; Tervonen, T.; Suominen, A.L.; Knuuttila, M.; Jula, A.; Ylöstalo, P. Association between Periodontal Condition and Hypertension in a Non-Smoking Population Aged 30-49 Years: Results of the Health 2000 Survey in Finland. *J. Clin. Periodontol.* **2014**, *41*, 1132–1138, doi:10.1111/jcpe.12316.

191. Zhu, Y.; Hollis, J.H. Associations between the Number of Natural Teeth and Metabolic Syndrome in Adults. *J. Clin. Periodontol.* **2015**, *42*, 113–120, doi:10.1111/jcpe.12361.

192. Hu, H.-Y.; Lee, Y.-L.; Lin, S.-Y.; Chou, Y.-C.; Chung, D.; Huang, N.; Chou, Y.-J.; Wu, C.-Y. Association Between Tooth Loss, Body Mass Index, and All-Cause Mortality Among Elderly Patients in Taiwan. *Medicine (Baltimore)* **2015**, *94*, e1543, doi:10.1097/MD.0000000000001543.

193. Choi, H.M.; Han, K.; Park, Y.-G.; Park, J.-B. Associations Among Oral Hygiene Behavior and Hypertension Prevalence and Control: The 2008 to 2010 Korea National Health and Nutrition Examination Survey. *J. Periodontol.* **2015**, *86*, 866–873, doi:10.1902/jop.2015.150025.

194. Laguzzi, P.N.; Schuch, H.S.; Medina, L.D.; de Amores, A.R.; Demarco, F.F.; Lorenzo, S. Tooth Loss and Associated Factors in Elders: Results from a National Survey in Uruguay. *J. Public Health Dent.* **2016**, *76*, 143–151, doi:10.1111/jphd.12123.

195. Kim, S.-W.; Cho, K.-H.; Han, K.-D.; Roh, Y.-K.; Song, I.-S.; Kim, Y.-H. Tooth Loss and Metabolic Syndrome in South Korea: The 2012 Korean National Health and Nutrition Examination Survey. *Medicine (Baltimore)* **2016**, *95*, e3331, doi:10.1097/MD.0000000000003331.

196. Singh, A.; Gupta, A.; Peres, M.A.; Watt, R.G.; Tsakos, G.; Mathur, M.R. Association between Tooth Loss and Hypertension among a Primarily Rural Middle Aged and Older Indian Adult Population. *J. Public Health Dent.* **2016**, *76*, 198–205, doi:10.1111/jphd.12136.

197. Moghadam, S.; Risbaf Fakour, S.; Ansari-Moghaddam, A.; Abdollahi, Z. A Relationship between Tooth Loss and Periodontal Disease with Increased Blood Pressure in Adults: A Population-Based Study in Iran. **2016**, *7*, 34–41.

198. Górski, B.; Nargiełło, E.; Grabowska, E.; Opolski, G.; Górska, R. The Association Between Dental Status and Risk of Acute Myocardial Infarction Among Poles: Case-Control Study. *Adv. Clin. Exp. Med. Off. Organ Wroclaw Med. Univ.* **2016**, *25*, 861–870, doi:10.17219/acem/58866.

199. Łysek, R.; Jankowski, P.; Polak, M.; Szafraniec, K.; Micek, A.; Wolfshaut-Wolak, R.; Łukaszewska, A.; Weber, T.; Czarnecka, D.; Pająk, A. Association between Central and Peripheral Blood Pressure and Periodontal Disease in Patients with a History of Myocardial Infarction. *Pol. Arch. Med. Wewn.* **2016**, *126*, 41–47, doi:10.20452/pamw.3265.

200. Kawabata, Y.; Ekuni, D.; Miyai, H.; Kataoka, K.; Yamane, M.; Mizutani, S.; Irie, K.; Azuma, T.; Tomofuji, T.; Iwasaki, Y.; et al. Relationship Between Prehypertension/Hypertension and Periodontal Disease: A Prospective Cohort Study. *Am. J. Hypertens.* **2016**, *29*, 388–396, doi:10.1093/ajh/hpv117.

201. Chrysanthakopoulos, N.A.; Chrysanthakopoulos, P.A. Association between Indices of Clinically-Defined Periodontitis and Self-Reported History of Systemic Medical Conditions. *J. Investig. Clin. Dent.* **2016**, *7*, 27–36, doi:10.1111/jicd.12119.

202. Muñoz-Torres, F.J.; Mukamal, K.J.; Pai, J.K.; Willett, W.; Joshipura, K.J. Relationship between Tooth Loss and Peripheral Arterial Disease among Women. *J. Clin. Periodontol.* **2017**, *44*, 989–995, doi:10.1111/jcpe.12787.

203. Aoyama, N.; Suzuki, J.-I.; Kobayashi, N.; Hanatani, T.; Ashigaki, N.; Yoshida, A.; Shiheido, Y.; Sato, H.; Kumagai, H.; Ikeda, Y.; et al. Periodontitis Deteriorates Peripheral Arterial Disease in Japanese Population via Enhanced Systemic Inflammation. *Heart Vessels* **2017**, *32*, 1314–1319, doi:10.1007/s00380-017-1003-6.

204. Im, S.I.; Heo, J.; Kim, B.J.; Cho, K.-I.; Kim, H.S.; Heo, J.H.; Hwang, J.Y. Impact of Periodontitis as Representative of Chronic Inflammation on Long-Term Clinical Outcomes in Patients with Atrial Fibrillation. *Open Heart* **2018**, *5*, e000708, doi:10.1136/openhrt-2017-000708.

205. Shin, H.-S. Association between the Number of Teeth and Hypertension in a Study Based on 13,561 Participants. *J. Periodontol.* **2018**, *89*, 397–406, doi:10.1002/JPER.17-0413.

206. Gordon, J.H.; LaMonte, M.J.; Genco, R.J.; Zhao, J.; Cimato, T.R.; Hovey, K.M.; Wactawski-Wende, J. Association of Clinical Measures of Periodontal Disease with Blood Pressure and Hypertension among Postmenopausal Women. *J. Periodontol.* **2018**, *89*, 1193–1202, doi:10.1002/JPER.17-0562.

207. Da, D.; Wang, F.; Zhang, H.; Zeng, X.; Jiang, Y.; Zhao, Q.; Luo, J.; Ding, D.; Zhang, Y.; Wu, B.; et al. Association between Tooth Loss and Hypertension among Older Chinese Adults: A Community-Based Study. *BMC Oral Health* **2019**, *19*, 277, doi:10.1186/s12903-019-0966-3.

208. Gordon, J.H.; LaMonte, M.J.; Zhao, J.; Genco, R.J.; Cimato, T.R.; Hovey, K.M.; Allison, M.A.; Mouton, C.P.; Wactawski-Wende, J. Association of Periodontal Disease and Edentulism With Hypertension Risk in Postmenopausal Women. *Am. J. Hypertens.* **2019**, *32*, 193–201, doi:10.1093/ajh/hpy164.

209. Qi, L.; Qian, Y.; Zhu, F.; Cao, N.; Lu, H.; Zhang, L. Association between Periodontal Disease and Tooth Loss and Mortality in an Elderly Chinese Population. *Aging Clin. Exp. Res.* **2020**, *32*, 2375–2382, doi:10.1007/s40520-019-01446-6.

210. Mendes, J.J.; Viana, J.; Cruz, F.; Pereira, D.; Ferreira, S.; Pereira, P.; Proença, L.; Machado, V.; Botelho, J.; Rua, J.; et al. Blood Pressure and Tooth Loss: A Large Cross-Sectional Study with Age Mediation Analysis. *Int. J. Environ. Res. Public. Health* **2021**, *18*, 285, doi:10.3390/ijerph18010285.

211. Woo, H.G.; Chang, Y.; Lee, J.S.; Song, T.-J. Tooth Loss Is Associated with an Increased Risk of Hypertension: A Nationwide Population-Based Cohort Study. *PloS One* **2021**, *16*, e0253257, doi:10.1371/journal.pone.0253257.

212. Hsu, P.-W.; Shen, Y.-W.; Syam, S.; Liang, W.-M.; Wu, T.-N.; Hsu, J.-T.; Fuh, L.-J. Patients with Periodontitis Are at a Higher Risk of Stroke: A Taiwanese Cohort Study. *J. Chin. Med. Assoc. JCMA* **2022**, *85*, 1006–1010, doi:10.1097/JCMA.0000000000000797.
